# Application of AI in Tablet Development: An Integrated Machine Learning Framework for Pre-Formulation Property Prediction

**DOI:** 10.3390/pharmaceutics18040452

**Published:** 2026-04-08

**Authors:** Masugu Hamaguchi, Tomoki Adachi, Noriyoshi Arai

**Affiliations:** 1 Department of Mechanical Engineering, Keio University, 3-14-1 Hiyoshi, Kohoku-ku, Yokohama 223-8522, Kanagawa, Japan; arai@mech.keio.ac.jp; 2 Kirin Central Research Institute, Kirin Holdings, 26-1, Muraoka-Higashi 2-Chome, Fujisawa 251-8555, Kanagawa, Japan; 3 Research Institute, Fancl Corporation, 12-13 Kamishinano, Totsuka-ku, Yokohama 244-0806, Kanagawa, Japan

**Keywords:** machine learning, tablet formulation, pre-formulation prediction, QSPR, pharmaceutics, particle size distribution

## Abstract

**Background/Objectives:** Tablet development requires simultaneous optimization of multiple quality attributes under limited experimental budgets, yet formulation–property relationships are highly nonlinear in mixture systems. To support pre-formulation decision-making prior to extensive tablet prototyping, this study proposes an AI framework that organizes formulation and process data together with raw-material property records into a reusable database, and enriches conventional composition/process features with physically motivated mixture descriptors derived from raw-material properties and formulation/process settings. **Methods:** Mixture-level scalar descriptors are constructed by composition-weighted aggregation of material properties, and particle size distribution (PSD) is incorporated via a compact set of summary statistics computed from composition-weighted mixture PSDs. Three feature sets are compared: (i) Materials + Processes (MP), (ii) MP with scalar Descriptors (MPD), and (iii) MPD with PSD summaries (MPDD). Five target properties are modeled: hardness, disintegration time, flow function, cohesion, and thickness. We train and evaluate Random Forest, Extra Trees Regressor, Lasso, Partial Least Squares, Support Vector Regression, and a multi-branch neural network that processes the three feature blocks separately and concatenates them for prediction. For interpolation assessment, repeated Train/Dev/Test splitting (5:3:2) across multiple random seeds is used, and the effect of feature augmentation is quantified by paired RMSE improvements with bootstrap confidence intervals and paired Wilcoxon signed-rank tests. To assess robustness under practical formulation updates, rolling-origin time-series splits are employed and Applicability Domain indicators are computed to characterize out-of-distribution coverage. **Results:** Across interpolation evaluations, mixture-descriptor augmentation (MPD/MPDD) improves hardness and disintegration time in most settings, whereas gains for flow function are smaller and cohesion/thickness show mixed effects under limited sample sizes. **Conclusions:** Under extrapolation-oriented evaluation, the descriptors can improve hardness but may degrade disintegration-time prediction under covariate shift, emphasizing the need for careful descriptor selection and dimensionality control when deploying pre-formulation predictors.

## 1. Background

In product development, multiobjective optimization to satisfy multiple target properties (specifications) simultaneously is commonplace; however, exploring candidate conditions requires iterative prototyping and experimentation, which imposes substantial time and cost burdens. In mixture systems in particular, interactions among raw materials often induce strongly nonlinear responses, making the search space high-dimensional and prone to nonconvexity.

In this study, we focus on tablets (compressed tablets) manufactured by compressing powders using a tableting machine. Tablets have been widely used not only in pharmaceuticals but also in dietary supplements because they can accurately contain a single dose and provide a stable, portable dosage form. However, tablet manufacturing requires fundamental powder properties, including flowability to ensure stable die filling and compressibility/compactability to reduce voids and form interparticle bonds during compression. Many active pharmaceutical ingredient powders exhibit poor tableting performance as-is, and it has been reported that fewer than 20% of active ingredients are suitable for direct compression [1]. Consequently, granulation-based approaches have been widely adopted to improve flowability and compressibility prior to tableting, including wet granulation using a binder solution and dry granulation in which primary particles are agglomerated under compressive stress [2]. In addition, advances in instrumentation and measurement technologies for tableting equipment have enabled monitoring and control of compaction pressure and fill depth, contributing to quality assurance and stable production. Furthermore, the development of compaction simulators that can reproduce production-scale compression cycles with small material quantities has facilitated understanding of compaction behavior and evaluation of scale-up [3].

From a formulation-design perspective, tablets comprise diverse excipients in addition to the active ingredient, such as fillers, binders, disintegrants, and lubricants; excipient design is essential both for achieving powder properties suitable for tableting and for ensuring manufacturability and tablet quality [4]. Moreover, improvements in flowability and compressibility and gains in productivity have been pursued through the development of high-functionality grades for direct compression, composite excipients, and the practical implementation of co-processed excipients [5,6]. In quality control, content uniformity is one of the most critical quality attributes and is strictly managed to ensure dose reproducibility and safety [7]. Thus, tablet development constitutes a complex system in which raw materials, composition, and process conditions interact, requiring design and process optimization to satisfy multiple properties simultaneously.

More broadly, tablet pre-formulation and early formulation/process development involve the integration of multiple factors, including active pharmaceutical ingredient (API) properties, excipient functionalities, particle size distribution, particle morphology, moisture-related properties, and processing conditions. These factors influence downstream critical quality attributes such as hardness, disintegration time, and thickness, as well as powder-processability attributes such as flowability and cohesion. Broadly, these variables can be categorized into material-related variables (e.g., composition, particle size distribution, physicochemical properties, and solid-state characteristics) and process-related variables (e.g., granulation conditions and compression settings). In this study, we focus on variables that are typically available in early-stage development datasets and use them to construct predictive models via mixture-level descriptor engineering.

In recent years, data-driven approaches, including machine learning and deep learning, have attracted attention as means to enhance design and decision making by learning patterns and relationships from large-scale data that are difficult for humans to identify. In materials science, research has advanced under the umbrella of materials informatics [8]; in chemistry and drug discovery, the QSAR/QSPR framework has been established within chemoinformatics, and descriptor-based prediction using numerical representations of structure and properties has been widely adopted [9]. In addition, the concept of process informatics, which links material properties and process conditions in an informatics framework for optimization, has been proposed [10]. For mixture systems such as tablets, appropriately aggregating component-level information into mixture-level features is important for both predictive performance and interpretability.

Regarding AI applications in tablet formulations, studies have reported learning the relationships between formulation/process conditions and critical quality attributes (CQAs) to predict properties such as hardness and disintegration time (DT). For example, Akseli et al. proposed a framework that combines non-destructive ultrasonic measurements with machine learning to estimate tablet fracture strength and disintegration behavior from tableting conditions and formulation factors [11]. In large-scale studies using curated formulation databases, deep neural networks and optimized ensemble models have demonstrated high predictive performance for DT and hardness, with some reports achieving $R^{2}>0.95$ [12,13].

However, many existing approaches incorporate post-compression properties, such as tablet hardness, friability, and wetting time, as input variables. Although such integration can improve predictive accuracy, it implicitly assumes that physical tablets have already been manufactured and characterized; consequently, its direct applicability to pre-manufacturing formulation screening and early-stage decision making is limited.

In contrast, efforts have also been reported to predict formulation-level properties using compositional descriptors and raw-material characteristics without relying on post-manufacturing measurements [14,15]. To enable prediction at the design stage, an appropriate representation of formulation mixtures is essential; however, many existing models do not explicitly model mixture-level physical agglomeration or particle size distribution (PSD) effects and instead directly encode excipient composition as a concentration vector.

In light of the above, this study introduces a feature-engineering strategy for mixture systems that considers (i) aggregation of physicochemical properties of raw materials according to mixing ratios and (ii) mixture properties that summarize mixture PSDs into statistically compact descriptors. By explicitly aligning feature construction with physical mixing behavior, we aim to strengthen predictive reliability at the true pre-formulation stage through controlled statistical comparisons and performance-improvement assessment using bootstrap confidence intervals. Furthermore, with an emphasis on prediction in extrapolative regions that is important in the context of process analytical technology (PAT) and quality by design (QbD), we quantify performance differences and coverage via stratified evaluation based on the applicability domain (AD) and assess extrapolation risk.

The objective of this study is to develop and validate a machine-learning framework for tablet development that enables pre-formulation screening using only raw-material information, composition, and process conditions, without relying on post-compression measurements as inputs. Accordingly, we address the following research questions (RQs). We compare three feature sets: MP (Materials + Processes: composition and process conditions only), MPD (MP plus composition-weighted scalar mixture descriptors), and MPDD (MPD plus PSD summary statistics from mixture particle-size distributions).

- **RQ1 (Performance gain by feature augmentation):** To what extent does augmenting MP with mixture descriptors and PSD summaries (MP→MPD/MPDD) improve predictive performance across target properties?
- **RQ2 (Robustness under deployment-like shift):** Under deployment-like temporal distribution shift (rolling-origin time-series split), are the improvements observed in interpolation evaluation preserved, and for which targets?
- **RQ3 (Risk screening via applicability domain):** Can applicability-domain (AD) indicators identify low-coverage regions where prediction errors increase, enabling AD-aware screening of risky predictions?

To answer these questions, the following section describes the dataset construction, feature-set design (MP/MPD/MPDD), model development, and evaluation protocols for both interpolation and extrapolation-oriented settings.

## 2. Materials and Methods

### 2.1. Sample Preparation and Measurement

The tablet samples comprised fillers, binders, disintegrants, lubricants, and other excipients. Examples of the materials used are shown in Table 1. Granulated materials were treated as raw materials in the same manner as primary powders. The data on formulations, process conditions, and powder/tablet properties used in this study were not obtained from public sources such as open databases; rather, all samples were prepared by the authors and all values were measured using the corresponding instruments. For sample preparation, each material was weighed using an analytical balance and uniformly blended according to the formulation. The blended powder was then filled and compressed using a single-punch tablet press for testing (N-30E, OKADA SEIKO, Okayama, Japan), and tablets were produced by compaction at the specified compaction force. The compaction force was set to 4400–19,600 N (450–2000 kgf).

In this study, we modeled five target variables relevant to tablet development, including post-compression tablet properties (hardness, disintegration time, and thickness) and pre-compression powder flow/cohesion characteristics. The definition and measurement method for each property are described below. These target variables are important indicators for evaluating tablet performance, quality, and manufacturability (including stability of filling and compression), and they are also key metrics in tablet design. Because the number of observations differs by target variable, the sample size is also provided for each.

- Hardness [N], n=1209: A measure of mechanical strength against external forces, directly related to chipping and capping during transport/handling and to edge defects and cracking during manufacturing. Excessive hardness, however, can impair disintegration and dissolution. Measured using a digital hardness tester (KHT-40N, FUJIWARA, Wakayama, Japan).
- Disintegration time [minute], n=882: The time required for a tablet to disintegrate under specified conditions, which particularly affects initial dissolution and absorption for immediate-release formulations. It is strongly influenced by the type and amount of disintegrant, lubricant level, hardness/compactness, PSD, and hydrophilic excipients. In this study, it was measured in water at 37 ℃ using a disintegration tester (NT-600, TOYAMA Industry, Toyama City, Japan).
- Flow function [-], n=151: A dimensionless index derived from shear-cell testing; higher values indicate better flowability. It is directly related to uniform die filling and to mass variability (a prerequisite for content uniformity). It varies with PSD, particle shape, surface roughness, moisture content, and electrostatic charging, as well as with the addition of lubricants and glidants. At the design stage, target flowability is ensured through the selection of granulation method and binder and through particle-size design, and is reflected in process settings such as hopper angle and feeder speed. Measured using a powder flow tester (Brookfield, Toronto, ON, Canada).
- Cohesion [kPa], n=145: A measure of the strength of adhesion/agglomeration between particles. High cohesion can promote bridging, rat-holing, classification, and segregation, thereby reducing flowability. It is sensitive to particle-size reduction, nonuniform moisture, surface energy, and electrostatic effects. In formulation design, cohesion is controlled via particle-size optimization, granulation, and addition of glidants to ensure stable compression behavior and uniform filling. Measured using a powder flow tester (Brookfield).
- Thickness [mm], n=60: Tablet thickness after compression reflects the fill mass and degree of compaction and affects swallowability, disintegration/dissolution behavior, and manufacturing stability. It correlates with weight and hardness, and changes in geometry can also alter drug diffusion behavior. Thickness depends on die/punch specifications and compression conditions; control within specification is also important for appearance conformity, packaging compatibility, and manufacturing stability. Measured using a micrometer (PK-1012CPX, Mitutoyo, Kawasaki, Japan).

Because sample size differs substantially across target variables, the results for the smaller datasets, particularly Flow function, Cohesion, and Thickness, should be interpreted as exploratory and with greater statistical uncertainty than those for Hardness and Disintegration Time. To clarify dataset structure, we additionally audited each target variable using formulation-level uniqueness defined on a material-composition basis. For the tablet-property targets Hardness, Disintegration Time, and Thickness, we also audited formulation–process-condition uniqueness defined on a combined material-composition and process-condition basis. Hardness (n=1209) comprised 388 unique formulations and 998 unique formulation–process conditions, and disintegration time (n=882) comprised 371 unique formulations and 698 unique formulation–process conditions. Thus, some observations shared identical formulation–process conditions in these two targets. Thickness contained repeated formulations (23 unique formulations across 60 observations) but all observations were unique once process conditions were included. For the pre-compression powder properties Flow function and Cohesion, uniqueness was evaluated on a material-composition basis only; under this definition, Flow function (n=151) comprised 136 unique formulations and Cohesion (n=145) comprised 134 unique formulations. Some observations originate from identical formulation–process conditions, and therefore the dataset is not fully independent. This is explicitly acknowledged as a limitation of the present study. Detailed counts are provided in the Supporting Information (Table S1).

### 2.2. Measurement of Raw-Material Powder Properties

To construct the descriptors described below, the physical and chemical properties of the raw materials were measured as follows.

- Loose bulk density: An indicator of powder packability used for evaluating powder flowability. Powder was poured into a 100 mL graduated cylinder, leveled, and the powder mass *m* was measured using an analytical balance. The bulk density was calculated as $\rho_{\mathrm{bulk}}=m/100\;\mathrm{mL}$.
- Tapped density: Bulk density after tapping. Powder was poured into a 100 mL graduated cylinder and manually tapped; the packed volume $V_{\mathrm{tap}}$ was read and the tapped density was calculated as $\rho_{\mathrm{tap}}=m/V_{\mathrm{tap}}$.
- Carr’s compressibility index: A flowability indicator based on the difference between the loose and tapped states.
  $$\mathrm{CI}=100\times \frac{\rho_{\mathrm{tap}}-\rho_{\mathrm{bulk}}}{\rho_{\mathrm{tap}}}$$
  Larger values indicate higher compressibility and tend to correspond to lower flowability.
- Hausner ratio: The density ratio of the tapped state to the loose state.
  $$\mathrm{HR}=\frac{\rho_{\mathrm{tap}}}{\rho_{\mathrm{bulk}}}$$
  Larger values indicate stronger interparticle interactions and tend to correspond to lower flowability.
- FLODEX: Using FLODEX (Hanson Research Corporation, Chatsworth, CA, USA), gravitational discharge from a specified orifice (whether discharge initiates and whether it is continuous) was evaluated and used as an indicator of powder flow behavior.
- Loss on drying: Calculated from the mass difference before and after heating at 80 ℃ for 20 min using HX204 (Mettler Toledo, Greifensee, Switzerland).
- Solubility score: An index based on visual observation of dissolution behavior in water under stirring, classified as soluble/poorly soluble/insoluble.
- Lipophilic score: An index classifying materials as lipophilic/non-lipophilic based on dissolution behavior.
- Flowability score: Based on single-material flowability evaluation (e.g., Flow function and FLODEX, Konya, Turkey) and prior formulation-development experience, an index classifying whether each material contributes to the flowability of a blended powder as “promoting,” “inhibiting,” or “neutral.”
- Particle size distribution: Measured using a laser diffraction particle size analyzer (LA-960, HORIBA, Chiyoda-ku, Tokyo) at a dispersion air pressure of 0.4 MPa. As distribution data, a frequency distribution corresponding to a common 80-point particle-size grid from 0.115 μm to 5000 μm was extracted.

### 2.3. Design of Additional Features

In this study, in addition to the single-material scalar properties (powder properties and scores) described above, we designed additional features to reflect formulation geometry, tableting conditions, and compositional balance in the mixture.

- R-part height: A shape parameter representing the contribution of the convex portion based on the tablet radius of curvature, intended to reflect differences in volume and density that cannot be captured by planar geometry alone.
- R-part volume: A proxy for the volume contribution of the convex portion derived from the R-part height and tablet diameter, intended to reflect the effect of shape differences on volume (and thus density and voids).
- 1/Weight: A reciprocal feature introduced to capture nonlinearity with respect to tablet weight (e.g., scale differences between small and large tablets).
- Compaction pressure: An indicator of pressure normalized by tablet area, intended to enable comparison of compressive stress intensity across tablets with different diameters.
- Raw-material category ratios (group sums): To reflect the mixture state, nine ratios normalized by tablet weight were calculated (microcrystalline cellulose ratio, disintegrant ratio, sugar-alcohol ratio, lubricant ratio, binder ratio, glidant ratio, granule ratio, main-component ratio, and other ratio).
- Second-order interactions (interaction-only): For seven of the above nine category ratios (from the microcrystalline cellulose ratio through the granule ratio), second-order interaction-only terms (without squared terms) were generated, adding a total of 21 interaction features.

## 3. AI Modeling Methods

**Figure 1:**
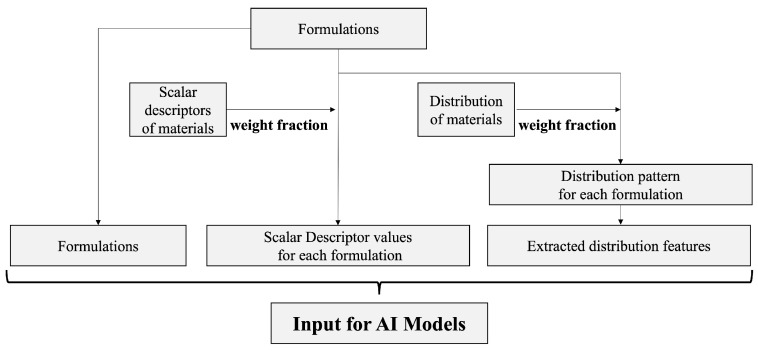
Workflow of AI modeling.

Figure 1 shows the modeling workflow.

### 3.1. Integration of Development Data and Database Construction

As a prerequisite for this study, formulation, process, and measurement data that had been managed in a distributed manner by individual researchers using separate Excel files were unified into a common format and reorganized into an internal database (DB) by building an automated ingestion pipeline (validation → transformation → storage) using robotic process automation (RPA). In parallel, a materials-properties database storing property information for the materials used was also created, and referential consistency between the two databases was ensured via a raw-material ID system. Operationally, RPA checks the completeness of required fields from the common template, normalizes units and notation variants, validates ID consistency, and detects duplicates; the data are then stored through an ETL pipeline in accordance with the schema. In addition to schema definitions, we established unit standardization, rules for handling missing values and duplicates, attribution metadata (product name, time period, equipment, operator, and version), and version control using update logs.

Hereafter, these databases are used as the single source for model training. As a secondary benefit of database construction, cross-researcher searching, sharing, and progress visualization became easier, leading to suppression of redundant experiments, more efficient leadership management, and improved reproducibility and traceability.

Although the DB integrates formulation, process, and measurement records for traceability and reuse, the predictive models in this study do not use any post-compression measurements as input variables. Instead, process and geometry features (e.g., compression pressure, tooling, target weight/geometry) are treated as design/process settings available prior to prototyping, and model inputs are limited to raw-material property records and formulation/process settings.

### 3.2. Descriptors

Powder property values measured in Section 2.2 were used as descriptors required for predicting tablet properties. However, while each descriptor is defined at the level of individual materials, the prediction target is a mixture. Therefore, mixture descriptors for each formulation were computed based on the following equation.

The purpose of this formulation is to enable estimation of property values even in situations where no physical mixture is available, such as in inverse design settings. Accordingly, for example, for disintegration-time prediction we do not use values that cannot be obtained without a physical sample, such as other target-variable values (e.g., hardness) or measured bulk density of the blended powder. Instead, without newly preparing and measuring mixture samples, we estimate mixture characteristics (mixture descriptors) from raw-material property values, blending ratios, and formulation/process settings, and use that information to predict tablet properties; this is one of the central aims of this study.

(1) $$p_{\mathrm{mix}}=\sum_{i=1}^{n}x_{i}\cdot p_{i}$$

Here, $p_{\mathrm{mix}}$ denotes the descriptor vector of the mixture, $x_{i}$ the mass fraction of component *i*, and $p_{i}$ the descriptor vector of component *i*, with the normalization condition $\sum_{i=1}^{n}x_{i}=1$. This formulation is intended as a practical approximation for constructing mixture-level features (mixture descriptors), rather than as a rigorous physical interaction model. In real powder mixtures, component interactions can be nonlinear; however, because sample size is limited for some target variables, introducing more complex nonlinear mixture descriptors would further increase descriptor dimensionality and the associated risk of overfitting. For example, vector information such as the particle size distribution addressed in this study can similarly be used to construct formulation-level distributions (mixture PSDs) based on blending ratios.

When descriptors are scalar, $p_{i}$ is a scalar value. In this study, the raw PSD data are vectors (80 dimensions), but this dimensionality is high relative to the sample size. Therefore, as described below, we first construct mixture PSDs based on blending ratios and then reduce dimensionality by converting them into summary features.

In contrast, all powder properties other than PSD are scalar. Among the scalar descriptors, missing values existed for some materials for reasons such as being unanalyzable or being legacy materials that could not be reanalyzed. Therefore, missing values were imputed using a *k*-nearest-neighbors imputer (k=5). The definitions of additional features assuming blended powders (e.g., raw-material category ratios) are as described in Section 2.3.

In this study, the set of descriptors used was varied by target variable. For example, basic powder properties such as loose bulk density, tapped density, Carr’s compressibility index, Hausner ratio, and loss on drying were included for all target variables. For hardness, disintegration time, and thickness, additional features reflecting the effects of geometry, density, and stress (R-part height/volume, 1/Weight, and compaction pressure) were added. For flow function and cohesion, FLODEX and the flowability score were added, and for disintegration time, the solubility score and lipophilic score were added. As a result, the dimensionality of the added scalar-descriptor block differed by target variable, ranging from 37 to 45 dimensions in this study (hardness: 44; disintegration time: 45; flow function: 37; cohesion: 37; thickness: 43). The full list of features used for each target is provided in the Supporting Information (Table S2).

For PSD, the material-level distribution vectors (80 dimensions) were combined by composition-weighted averaging according to Equation 1 to construct a mixture PSD for each formulation. For materials with missing PSD information, the distribution vector was treated as a zero vector so that it did not contribute to the formulation distribution, and the mixture PSD was then normalized such that the total contribution of non-missing materials summed to 100%. Subsequently, to reduce overfitting risk, dimensionality was reduced by converting the 80-dimensional mixture PSD into summary features (to 10 dimensions). Specifically, we used quantiles ($d_{10}$, $d_{50}$, $d_{90}$), mean particle size, standard deviation, skewness, kurtosis, the fraction of particles ≤25μm, the fraction of particles ≥150μm, and entropy as features. This summary-feature transformation was adopted as a practical measure to retain distributional information while mitigating the high-dimensionality problem in MPDD.

### 3.3. Dataset Construction

To evaluate whether the descriptors described above improve predictive performance, we constructed the following three datasets. First, we defined MP (Materials + Processes) as a 157-variable *base* feature set by concatenating material addition rates and process information (e.g., compaction force, punch type, and tablet diameter). This 157-variable count is defined *before* one-hot encoding; because punch type is one-hot encoded in preprocessing (K=2 in our dataset), the numeric MP input replaces the single punch-type column with *K* dummy columns, yielding 151 (material addition rates) + 5 (process variables) + 2 (punch-type dummies) =158 input dimensions for MP. Next, we defined MPD (Materials + Processes + Descriptors) by adding the scalar-descriptor block described in Section 3.2 (37–45 dimensions depending on the target variable) to MP. Finally, we defined MPDD (Materials + Processes + Descriptors + Distribution) by adding summary features derived from PSD (10 dimensions) to MPD. Thus, material composition and process variables are included directly in MP, whereas composition-weighted aggregation is used specifically to construct mixture-level scalar descriptors and mixture PSDs from material-level information. The procedures for PSD mixing/normalization and transformation to summary features are as described in Section 3.2.

### 3.4. Model Development

#### 3.4.1. Model Selection

Although the dimensionality of the full feature set (including material addition rates, descriptors, and PSD information) differs by target variable, it becomes high-dimensional (generally in the 200 s for MPDD). Therefore, we applied machine learning and deep learning to learn predictive relationships from these many features. Because all target variables in this study are continuous, we used supervised learning for regression. The input explanatory variables are the datasets described in Section 3.3, and the target variables are the evaluation indices relevant to tablet development described in Section 2.1 (post-compression tablet properties: hardness, disintegration time, and thickness; and pre-compression powder properties: cohesion and flow function). In addition to the PSD summarization described above, we addressed the high-dimensional setting by including models with dimensionality-control or regularization properties (Lasso and PLS), by comparing tree-based models that can provide implicit feature selection, and by evaluating performance over repeated random splits rather than relying on a single partition.

**Figure 2:**
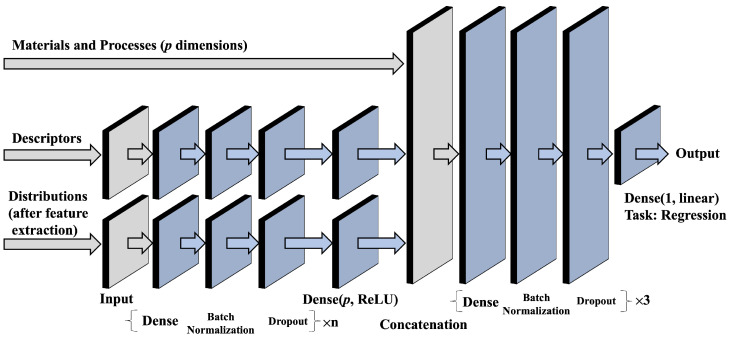
Architecture of the deep learning model.

For machine learning, we employed two tree-based models (Random Forest Regressor (RF) and Extra Trees Regressor (ET)), two linear models (Lasso and Partial Least Squares (PLS)), and one support-vector-based model (Support Vector Regressor (SVR)). We also used a deep learning model (Neural Network, NN) designed to input the data patterns described in Section 3.3 into separate branches (see Figure 2). We introduced this NN because we expected that a multi-branch architecture could flexibly model heterogeneous feature blocks and potentially improve predictive performance by learning interactions within and across these blocks. Specifically, we prepared three input branches: material and process information (157 base variables; 158 input dimensions after one-hot encoding punch type), scalar-descriptor information (37–45 dimensions depending on the target variable), and PSD-derived summary features (10 dimensions).

In this architecture, for PSD we first reduced dimensionality by converting the mixture PSD to summary features and then processed them through a dedicated neural network layer for that input branch to account for interactions among the PSD-derived features. Similarly, the scalar-descriptor set was processed through a dedicated layer for that branch to extract interaction features among scalar variables. Each input branch was processed through independent neural network layers; the resulting representations were then concatenated with the material and process data (“Materials and Processes” in Figure 2) and passed through additional neural network layers to predict the target variable.

For both machine learning and deep learning, we independently built models for each target variable to obtain prediction models optimized for each property. Among the explanatory variables, formulation ratios (material addition rates) were not standardized, whereas other numerical features (process conditions, scalar descriptors, and PSD-derived summary features) were standardized to mean 0 and standard deviation 1. PSD summary features were computed from mixture PSDs normalized so that each sample (row) summed to 1 and were treated as scalar features in the same manner as other continuous variables. To avoid data leakage, all preprocessing operations that require fitting to the data distribution (e.g., standardization of numerical variables and one-hot encoding of categorical variables) were fitted using the Train split only within each split and then applied to Dev/Test. For analyses that require fitting on Train+Dev within a fold (e.g., AD thresholds and PCA-based dimensionality estimation in the extrapolation setting), fitting was restricted to Train+Dev of that fold; inference (evaluation) was then performed on Test only.

For interpolation prediction described below, we used all target variables and models listed above. For extrapolation prediction in the main text, we focus on hardness and disintegration time; time-split results for other targets (flow function, cohesion, and thickness) are provided in the Supplementary Information (Tables S15–S17). In addition, in the extrapolation comparison under the rolling-origin setting (equivalent to 5 folds; see Section 3.7.1), NN was excluded because training variability was large and it was inferred to produce outlier evaluation values under a small number of folds.

#### 3.4.2. Hyperparameter Optimization

For hyperparameter optimization of the machine learning models, the hyperparameter ranges for each model are shown in Table 2. Hyperparameters were optimized using grid search. In contrast, because hyperparameter tuning for the deep learning model is computationally expensive, we applied Bayesian optimization (Optuna) to search for configurations that minimize validation loss (RMSE). The types of hyperparameters and their search ranges for deep learning are shown in Table 3. In each trial, a model was generated based on the same preprocessing and feature configuration and trained using early stopping and learning-rate decay based on validation loss; the validation RMSE was evaluated as the objective function value. The number of Optuna trials was set to 50 while confirming convergence of the loss values.

### 3.5. Model Interpretation (SHAP Analysis)

In this study, for models that achieved practically sufficient accuracy in interpolation prediction, we adopted SHAP (SHapley Additive exPlanations) to quantify the contribution of each feature to predictions. SHAP is a game-theory-based approach that explains machine learning model outputs by assigning an importance value to each feature [16]. We computed SHAP values for the trained ET (Extra Trees Regressor) model (feature set: MPDD) and used the mean absolute SHAP value, Mean(|SHAP|), as the feature-importance measure. To reduce the influence of random data splitting, we summarized importance by averaging results across repeated experiments conducted with 50 random seeds. To mitigate the effect of feature scaling, preprocessing during SHAP computation was aligned with that used in model training.

### 3.6. Evaluation Design for Interpolation Prediction

#### 3.6.1. Data Splitting

Interpolation prediction was designed to evaluate generalization performance within the observed design space and the pure effect of using descriptors without the influence of covariate shift. For accuracy evaluation of both machine learning and deep learning models, we adopted an evaluation procedure that emphasizes appropriate data splitting and reproducibility. For interpolation evaluation, the full dataset was split into Train/Development (Dev)/Test at a ratio of 50%:30%:20%. Models were trained on the Train set, validated and tuned on the Dev set, then retrained on the combined Train+Dev set before final generalization performance was assessed on the Test set. To reduce dependence on any specific random split, we conducted repeated experiments (n=50) while varying the split random seed, and used the mean across all trials as the evaluation metric.

#### 3.6.2. Performance Metrics

As performance metrics, we used the coefficient of determination ($R^{2}$) and the root mean squared error (RMSE). $R^{2}$ evaluates the strength of association between predicted and measured values, whereas RMSE evaluates the absolute magnitude of prediction error. These are defined as follows.

(2) $$\begin{array}{cc} R^{2} & =1-\frac{\sum_{i=1}^{n}{\left(y_{i}-{\hat{y}}_{i}\right)}^{2}}{\sum_{i=1}^{n}{\left(y_{i}-\bar{y}\right)}^{2}}\\ \mathrm{RMSE} & =\sqrt{\frac{1}{n}\sum_{i=1}^{n}{\left(y_{i}-{\hat{y}}_{i}\right)}^{2}} \end{array}$$

Here, $y_{i}$ denotes the measured value, ${\hat{y}}_{i}$ the predicted value, $\bar{y}$ the mean of the measured values, and *n* the sample size.

#### 3.6.3. Statistical Analysis of Effect Sizes

In this study, using RMSE on the Test set as the primary metric, we examined the effectiveness and reproducibility of feature augmentation by descriptors described in Section 3.2. First, under the same conditions (same learning model and same data split), we computed the difference between the RMSE obtained with the baseline (MP) and the RMSE obtained with the explanatory variables augmented with descriptors (MPD or MPDD):

d=RMSE(MP)−RMSE(MPDorMPDD)

Here, d>0 indicates a reduction in RMSE (improved predictive accuracy) due to feature augmentation.

As a summary of effect sizes, for each feature set we computed the mean improvement $\bar{d}$ by learning model and its 95% bootstrap confidence interval (CI). The bootstrap resampling unit was the paired result obtained under the same model and the same seed; we used a paired bootstrap with replacement (percentile method; number of resamples B=50,000) and obtained ${\mathrm{CI}}_{95\%}=\left({\mathrm{CI}}_{\mathrm{low}},{\mathrm{CI}}_{\mathrm{high}}\right)$ for the mean difference. In addition, as a supplementary test-based evaluation, we performed a Wilcoxon signed-rank test (two-sided, p<0.05) for each set of {d}. Given the exploratory nature of this supplementary test, *p*-values are reported without multiplicity correction; effect sizes and confidence intervals are emphasized.

To evaluate reproducibility of the effect-size summary (mean improvement), we partitioned the 50 random seeds (the 50 repeats described in Section 3.6.1) into 25 non-overlapping *discovery* seeds and 25 *validation* seeds. In the discovery set, for each (target variable, feature set, learning model) combination, we computed the mean improvement ${\bar{d}}^{\left(\mathrm{disc}\right)}$ across 25 seeds, and then computed the corresponding mean improvement ${\bar{d}}^{\left(\mathrm{valid}\right)}$ from the validation set. We judged “directional agreement” when $\mathrm{sign}({\bar{d}}^{\left(\mathrm{disc}\right)})=\mathrm{sign}({\bar{d}}^{\left(\mathrm{valid}\right)})$. The directional-agreement rate was computed as r=k/n, where *n* is the number of (target variable, feature set, learning model) combinations and *k* is the number with agreement. In this study, n=60 (5 target variables × 2 feature sets × 6 learning models). For confidence intervals, we used the 95% Clopper–Pearson confidence interval.

### 3.7. Evaluation Design for Extrapolation Prediction

#### 3.7.1. Data Splitting

To mimic temporal distribution shift encountered in practice, we conducted extrapolation-oriented evaluation using rolling-origin time-series splits. As a premise, formulation data in this study are ordered in time; however, product renewals or new product development can introduce new materials or material combinations not present in past datasets. Therefore, we considered that extrapolation prediction can be evaluated by training on past data and predicting future data.

For splitting extrapolation data, based on a rolling-origin strategy we used an expanding-window scheme in which the Train window cumulatively expands forward in time with a fixed start point, and the subsequent Development (Dev) and Test windows are evaluated with fixed widths to reduce dependence of performance on a particular split. Specifically, we set the initial split into Train/Development (Dev)/Test at a ratio of 50%:20%:10%. The remaining 20% was advanced in four steps with a step width of 5%, yielding 5 folds. This design is not a simple time-series split with fixed ratios (e.g., 60/20/20) but a rolling-origin (expanding-window) scheme defined by an initial window and a step size.

#### 3.7.2. Definition of Applicability Domain (AD)

In extrapolation prediction, to quantitatively verify whether the Test data defined by rolling-origin splitting are extrapolative, we adopted the following four applicability domain (AD) indicators. All AD thresholds (including PCA-based effective dimensionality estimation for MD) were fitted within each fold using Train+Dev only, and then applied to Dev/Test.

- Leverage: Based on the hat matrix of a linear model, representing the degree of extrapolation of the test data relative to the training data (Train+Dev). The 95th-percentile threshold of hat values in Train+Dev was used as the criterion for extrapolation.
- Mahalanobis distance (MD): The effective dimensionality was estimated by PCA, and extrapolation was judged using a threshold based on the chi-square distribution for squared distances.
- Mean *k*-nearest-neighbors distance (kNN): Extrapolation was judged using the 95th percentile of the mean distance to the *k* nearest neighbors in Train+Dev as the threshold.
- Range OK: Percentage of samples within the per-feature Train+Dev minimum–maximum bounds.

#### 3.7.3. Performance Metrics

In interpolation evaluation, we used $R^{2}$ and RMSE as performance metrics; in extrapolation evaluation, we additionally evaluated rank correlation (Spearman’s ρ), defined as follows.

(3) $$\begin{array}{cc} R^{2} & =1-\frac{\sum_{i=1}^{n}{\left(y_{i}-{\hat{y}}_{i}\right)}^{2}}{\sum_{i=1}^{n}{\left(y_{i}-\bar{y}\right)}^{2}}\\ \mathrm{RMSE} & =\sqrt{\frac{1}{n}\sum_{i=1}^{n}{\left(y_{i}-{\hat{y}}_{i}\right)}^{2}} \end{array}$$

(4) $$\rho \;=\;\mathrm{corr}\left(\mathrm{rank}\left(y\right),\;\mathrm{rank}\left(\hat{y}\right)\right)$$

Here, $y_{i}$ denotes the measured value, ${\hat{y}}_{i}$ the predicted value, $\bar{y}$ the mean of the measured values, and *n* the sample size. In addition, rank(y) and $\mathrm{rank}(\hat{y})$ denote the ranks assigned to the measured-value vector $y=(y_{1},\ldots ,y_{n})$ and the predicted-value vector $\hat{y}=\left({\hat{y}}_{1},\ldots ,{\hat{y}}_{n}\right)$, respectively (ties are assigned average ranks), and corr(·,·) denotes the Pearson correlation coefficient. When there are no ties, letting $d_{i}=\mathrm{rank}\left(y_{i}\right)-\mathrm{rank}\left({\hat{y}}_{i}\right)$ yields Equation (5).

(5) $$\rho \;=\;1\;-\;\frac{6\sum_{i=1}^{n}d_{i}^{2}}{n\;\left(n^{2}-1\right)}$$

## 4. Results and Discussion

### 4.1. Model Performance in Interpolation Prediction

#### 4.1.1. Overall Performance Comparison

**Figure 3:**
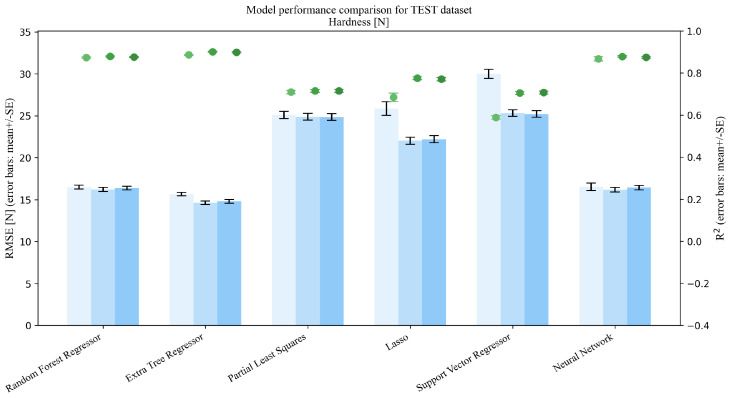
Comparison of model performance on the test dataset (Hardness, n=1209).

**Figure 4:**
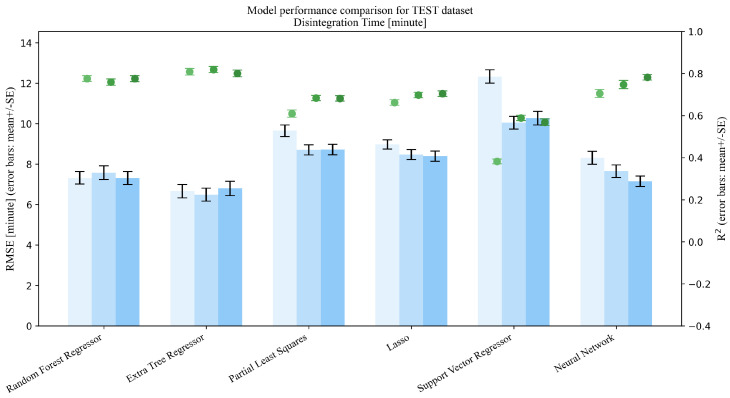
Comparison of model performance on the test dataset (Disintegration time, n=882).

**Figure 5:**
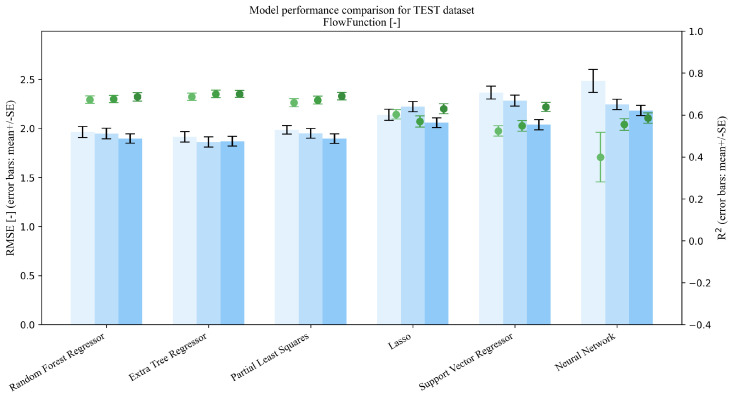
Comparison of model performance on the test dataset (Flow function, n=151).

**Figure 6:**
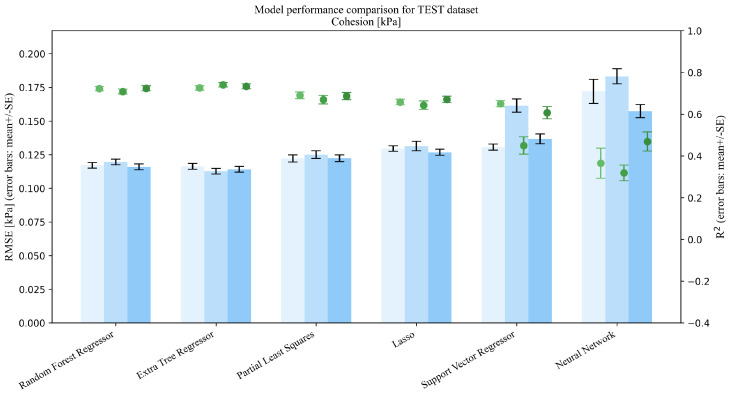
Comparison of model performance on the test dataset (Cohesion, n=145).

**Figure 7:**
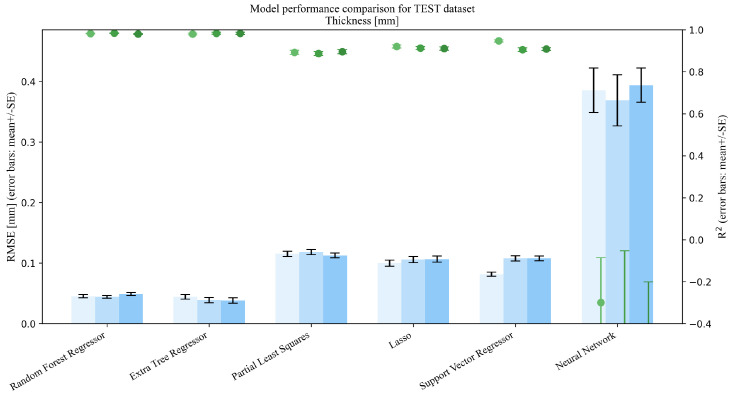
Comparison of model performance on the test dataset (Thickness, n=60).

**Figure 8:**
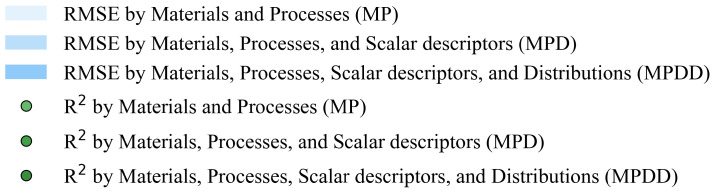
Legend for dataset patterns.

**Figure 9:**
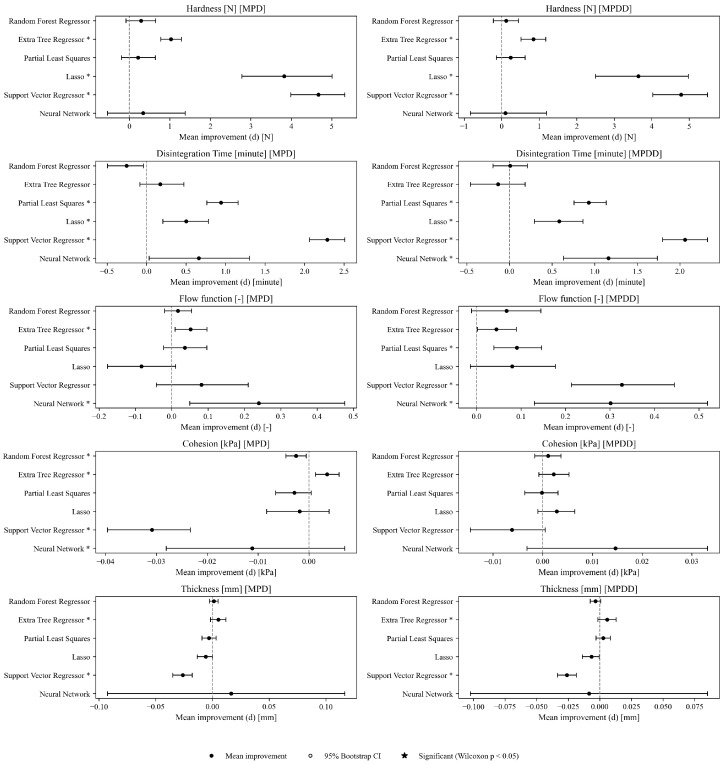
Forest plot of RMSE improvement d=RMSE(MP)−RMSE(MPD/MPDD). Each panel indicates a target variable × feature set (MPD or MPDD). Points denote the mean improvement $\bar{d}$, and horizontal lines denote the 95% bootstrap CI (percentile method, B=50,000). The vertical dashed line indicates d=0 (no improvement). “*” indicates p<0.05 in the two-sided Wilcoxon signed-rank test. Sample sizes are n=1209 (hardness), n=882 (disintegration time), n=151 (flow function), n=145 (cohesion), and n=60 (thickness).

This section addresses Research Question 1 (RQ1), which concerns how the addition of mixture descriptors (MP→MPD) and PSD summaries (MP→MPDD) affects model performance under repeated random splits (interpolation setting). Dual-axis performance comparisons ($R^{2}$ and RMSE) on the Test set are shown in Figure 3, Figure 4, Figure 5, Figure 6 and Figure 7. Each figure compares the baseline MP (Materials + Processes) with feature sets augmented by scalar descriptors (MPD: MP + Descriptors) and further augmented by PSD summaries (MPDD: MPD + Distribution) in a consistent format (see Figure 8 for the legend). Additional representative parity plots for the best models are provided in the Supporting Information (Figures S1–S5). In addition, we evaluated the significance of RMSE improvements relative to MP using percentile bootstrap CIs and Wilcoxon signed-rank tests, and present the results as a forest plot in Figure 9. Finally, for interpretation of effects, we summarize improvements relative to MP in Table 4 and compare performance of the *best model* for each target variable in Table 5. For each target variable, among all combinations of the six learning models and three feature sets (MP/MPD/MPDD), we define the “*best model*” as the combination (algorithm + feature set) that yields the smallest mean Test RMSE across the 50 repeated random splits.

#### 4.1.2. Statistical Analysis of Performance Improvements

We estimated the RMSE improvement relative to MP, d=RMSE(MP)−RMSE(MPD/MPDD), and evaluated significance based on percentile bootstrap CIs (B=50,000) and a two-sided Wilcoxon signed-rank test. The results are shown as a forest plot in Figure 9.

Overall, for hardness, many models show a positive mean improvement $\bar{d}$ for both MPD and MPDD, and for multiple models the 95% bootstrap CI does not cross 0. In addition, some combinations fall below the significance threshold based on uncorrected Wilcoxon *p*-values computed as exploratory Supplementary Information. For disintegration time, MPD shows positive improvements for PLS/Lasso/SVR, whereas RF yields a negative mean improvement, indicating remaining model dependence. For MPDD, positive improvements are observed for Lasso/NN/PLS/SVR, whereas RF shows nearly no improvement and ET shows a slightly negative mean improvement, indicating variability in improvement. For flow function, effect sizes are small, but MPDD shows positive improvements for NN/PLS/SVR, and a similar tendency is suggested by exploratory uncorrected *p*-values. For cohesion, MPD exhibits a mix of improvement and degradation, with negative improvements observed for some models; in contrast, MPDD shows neither clear improvement nor degradation, leaving uncertainty in the effect. Thickness has the smallest sample size and thus unstable estimates; because both improvement and degradation are observed, caution is required when generalizing effects.

In both the discovery set and the validation set, the sign of $\bar{d}$ agreed for k=50 combinations (out of a total of n=60 combinations), yielding a directional-agreement rate of r=0.833 (95% Clopper–Pearson CI: 0.715–0.917). This suggests that the direction of the effect is reproducible across random splits.

#### 4.1.3. Performance Improvement by Descriptors

Finally, we summarize performance improvements relative to MP in Table 4 and compare performance for the best model for each target variable in Table 5. Table 4 aggregates results across the six learning models; the MP performance represents the mean performance under MP across all models for each target variable. In contrast, Table 5 reports mean metrics restricted to the best model for each target variable; therefore, the values in the two tables do not generally coincide. As shown in Table 4, for hardness, disintegration time, and flow function, both MPD and MPDD show average improvements, with the improvement being larger for flow function under MPDD. In contrast, for cohesion, MPD shows average degradation while MPDD shows only a slight improvement, and for thickness no improvement is observed for either MPD or MPDD. Moreover, when restricted to the best model (Table 5), some target variables can lead to conclusions that differ from those based on aggregated improvements (e.g., MPDD underperforms MP for disintegration time). Therefore, for target-specific use, both tables should be interpreted together. Across targets, NN sometimes benefited from descriptor augmentation, but its advantage was not as clear or consistent as that of the tree-based models in the present dataset. Accordingly, the main empirical interpretation below emphasizes the more stable findings obtained from ET/RF-based comparisons.

For hardness, the best-performing model under the interpolation setting achieved a mean test RMSE of 14.577 N with $R^{2}=0.901$; given the observed hardness range in this dataset (7.93–374.78 N), this corresponds to approximately 4.0% of the observed range. For disintegration time, the best-performing model achieved a mean test RMSE of 6.487 min with $R^{2}=0.819$, corresponding to approximately 5.4% of the observed range (0.39–120.00 min); for flow function, RMSE was 1.861 with $R^{2}=0.700$, corresponding to approximately 11.2% of the observed range (2.655–19.28); for cohesion, RMSE was 0.113 with $R^{2}=0.741$, corresponding to approximately 10.8% of the observed range (0.184–1.233); and for thickness, RMSE was 0.038 mm with $R^{2}=0.983$, corresponding to approximately 3.1% of the observed range (4.17–5.41 mm).

#### 4.1.4. Feature Contributions Based on SHAP Analysis

**Figure 10:**
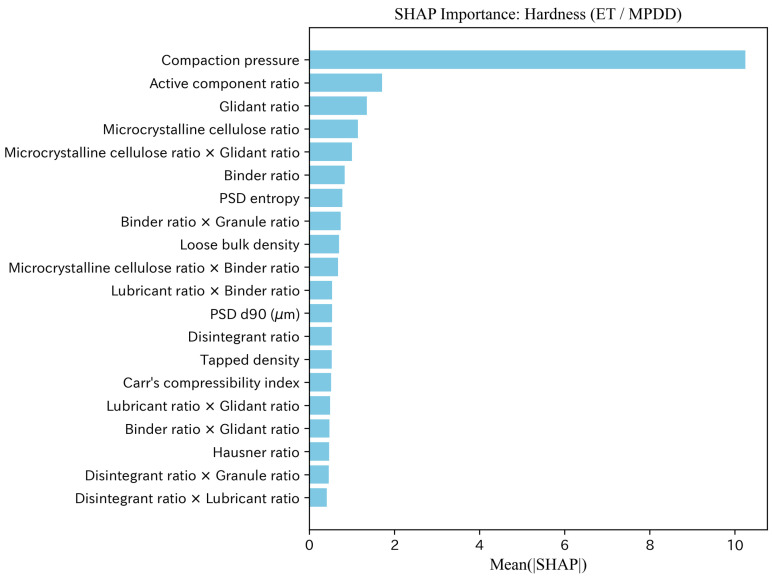
SHAP feature importance (Mean(|SHAP|)): Hardness (ET/MPDD).

**Figure 11:**
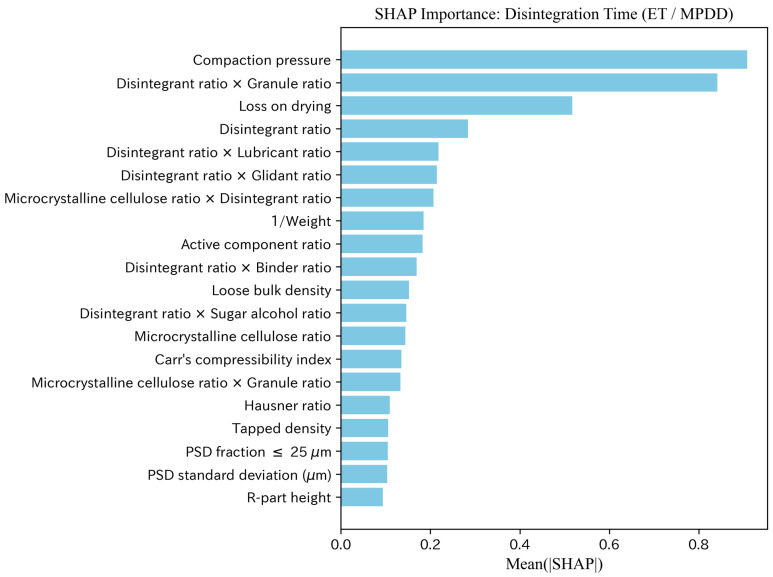
SHAP feature importance (Mean(|SHAP|)): Disintegration time (ET/MPDD).

We performed SHAP analysis for the best model (ET) in the interpolation evaluation and summarized feature contributions using Mean(|SHAP|). Representative SHAP importance plots for Hardness and Disintegration time are shown in Figure 10 and Figure 11, and additional target-specific SHAP visualizations for Flow function, Cohesion, and Thickness are provided in the Supporting Information (Figures S6–S8). To facilitate deeper discussion of feature meanings, SHAP analysis was performed consistently under the MPDD setting regardless of comparative predictive accuracy.

For hardness, compaction pressure was dominant, followed by category ratios such as main-component ratio, glidant ratio, and microcrystalline cellulose ratio, as well as powder properties (e.g., Hausner ratio and PSD entropy) and interaction terms. For disintegration time, in addition to compaction pressure, “disintegrant ratio × granule ratio” and loss on drying ranked highly, suggesting contributions from both moisture/composition and tableting conditions. For flow function, PSD fraction ≤25μm ranked highest, and PSD-related information such as the glidant ratio and quantiles (PSD d10 (μm) and PSD d50 (μm)) occupied the upper ranks. For cohesion, PSD fraction ≤25μm and PSD d50 (μm) also ranked highly; however, the absolute magnitudes of importance were small overall, leaving uncertainty in the effect. For thickness, compaction pressure and 1/Weight were the primary factors, and formulation ratios (e.g., glidant ratio) also showed non-negligible contributions.

### 4.2. Model Performance in Extrapolation Prediction

#### 4.2.1. Overall Performance Comparison

This section addresses Research Question 2 (RQ2), which concerns whether improvements under interpolation are preserved under deployment-like temporal distribution shift (rolling-origin time-series split), and Research Question 3 (RQ3), which concerns whether applicability-domain (AD) indicators can identify low-coverage regions where prediction errors increase and enable AD-aware screening of risky predictions.

#### 4.2.2. Performance Improvement by Descriptors

Using MP as the baseline, we summarize performance changes due to adding descriptors (MPD) and PSD summaries (MPDD) as “performance improvements.” Here, improvements in $R^{2}$ and Spearman’s ρ are defined as $R^{2}\left(\mathrm{MPD}/\mathrm{MPDD}\right)-R^{2}\left(\mathrm{MP}\right)$ and ρ(MPD/MPDD)−ρ(MP), respectively, and improvement in RMSE is defined as RMSE(MP)−RMSE(MPD/MPDD); in all cases, positive values indicate improvement.

As an aggregated summary across multiple models, we computed improvements for the five models excluding NN (RF/ET/Lasso/PLS/SVR) and summarized them by their mean; the results are shown in Table 6.

As an additional supplementary evaluation, we fixed the best model under MP (Hardness: RF; Disintegration time: Lasso) and compared the metrics across feature sets (MP/MPD/MPDD) using the same model; the results are shown in Table 7.

In both the aggregated summary across models and the supplementary evaluation with the best model fixed, hardness shows $\Delta R^{2}>0$, RMSE improvement >0, and Δρ>0, indicating that adding descriptors/distribution features tends to improve performance even under extrapolative conditions. In contrast, for disintegration time, $\Delta R^{2}$ and Δρ are negative, suggesting reduced monotonicity in ranking. The RMSE improvement is also negative on average, indicating an overall tendency toward degradation.

#### 4.2.3. AD-Related Diagnostics and Error Analysis

**Figure 12:**
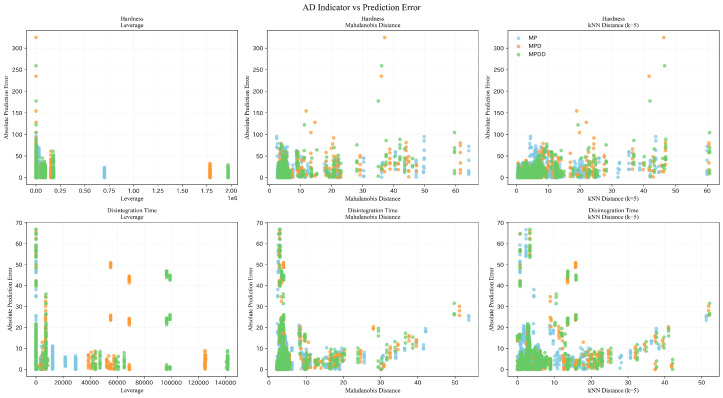
Scatter plots of applicability domain (AD) indicators versus absolute prediction error under rolling-origin time-series split (top: hardness, bottom: disintegration time; colors: MP in blue, MPD in orange, and MPDD in green).

Table 8 compares AD coverage under rolling-origin time-series splitting for extrapolation evaluation. For each target variable × feature set (MP/MPD/MPDD), we summarized fold-averaged Train+Dev/Test coverage for Leverage (Lev), Mahalanobis distance (MD), kNN, and the proportion within the training range (Range OK; within the minimum–maximum range of each feature). To address Research Question 3 (RQ3), i.e., whether AD indicators can identify low-coverage regions with increased prediction errors, Figure 12 presents scatter plots relating AD indicator values to absolute prediction errors for hardness and disintegration time across all test samples and folds.

As shown in Table 8, AD coverage on the Train+Dev side is generally high across all indicators. In contrast, leverage decreases on the Test side (e.g., Lev: 43.8% for Hardness/MP, 23.1% for Hardness/MPD, and 40.2% for Disintegration Time/MPD), suggesting that a non-negligible fraction of future data lies near the boundary of the training space. Meanwhile, MD and Range OK remain high even on the Test side, indicating that sensitivity to extrapolation differs among AD indicators.

Figure 12 shows the relationship between three AD indicators and absolute prediction errors aggregated over all rolling-origin folds (columns: leverage, Mahalanobis distance, and kNN distance with k=5). Larger indicator values correspond to test samples located farther from the training distribution (computed per fold). Overall, the association between AD indicators and errors is weak and heterogeneous. For hardness, leverage does not exhibit a strictly monotonic relationship with error, but the distance-based indicators (Mahalanobis and kNN distance) show a modest rise in the upper envelope of errors at larger distances. For disintegration time, substantial errors are also observed at small AD values, suggesting that AD indicators alone do not fully explain extrapolation difficulty. These results motivate AD-aware screening as a complementary risk flag for deployment under distribution shift, to be used alongside empirical calibration and ongoing error monitoring.

#### 4.2.4. Factors Underlying the Differential Extrapolation Performance

**Figure 13:**
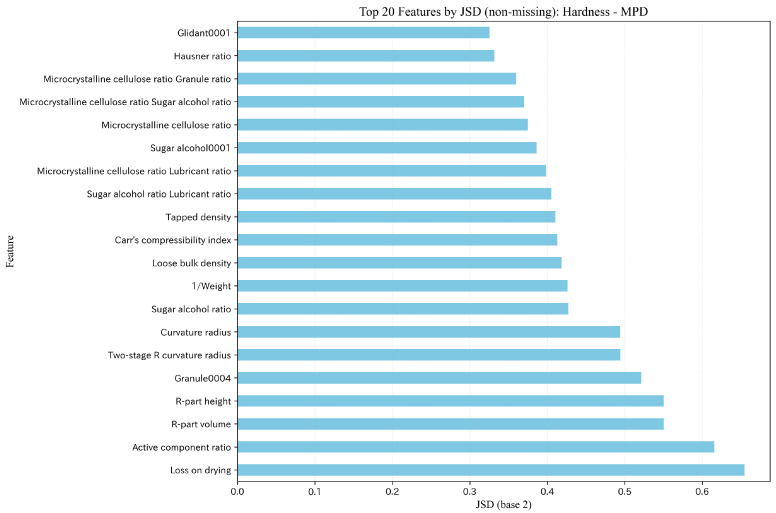
Top 20 features by JSD (non-missing) under the rolling-origin split (Hardness, MPD).

**Figure 14:**
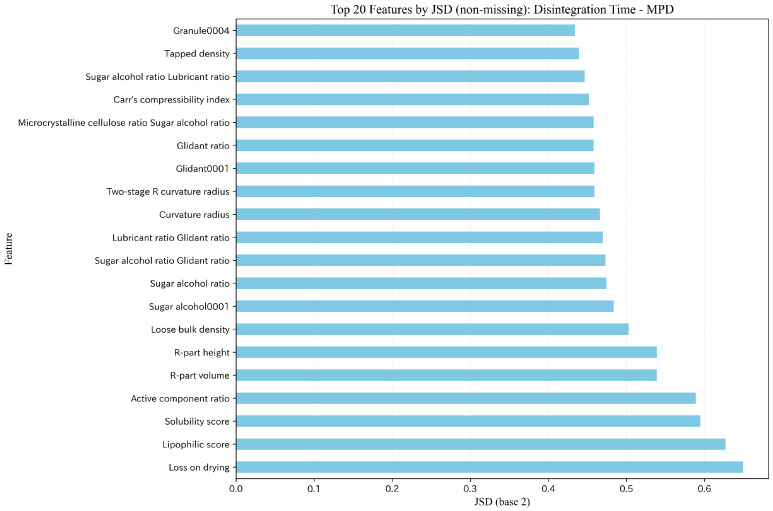
Top 20 features by JSD (non-missing) under the rolling-origin split (Disintegration time, MPD).

Under rolling-origin time-series splitting, the covariate distribution can shift between the training period (Train+Dev) and the future period (Test). We quantified covariate shift using Jensen–Shannon divergence (JSD) on non-missing feature distributions and observed that feature augmentation (MP → MPD/MPDD) tends to make distributional differences more pronounced. We selected JSD as the primary indicator because it is symmetric between train/test distributions, bounded for stable cross-target comparison, and less prone to inflation under zero-dominated feature patterns than binning-sensitive indices. As a compact summary, the mean JSD of the top-10 shifted features (mean JSD (Top 10)) was 0.380/0.540/0.584 for Hardness under MP/MPD/MPDD and 0.409/0.576/0.629 for Disintegration time. These values were obtained by computing feature-wise JSD per rolling-origin fold (Train+Dev vs Test) and then averaging JSD across folds for each feature; the top-10 features were ranked by this fold-averaged JSD, and the reported mean is the mean of the top-10 fold-averaged JSD values. Figure 13 and Figure 14 show representative top-20 shifted features under MPD. In this dataset, the missing-rate shift component was negligible, and the observed shift was primarily driven by changes in the non-missing feature distributions. Implementation details (binning, fold aggregation, smoothing, missing/zero handling, and log base) are provided in the Supporting Information; additional JSD/PSI summaries are shown in Figures S9–S14 and Table S18. The key question is whether the input–output mapping (X→y) remains sufficiently stable under the observed shift.

For hardness, dominant drivers such as compaction pressure, geometric scale (e.g., weight), and category ratios tend to retain consistent directional effects across formulation updates. Therefore, adding descriptors and PSD summaries can complement information relevant to packing, effective bonding area, and contact mechanics, thereby improving generalization even under covariate shift. Consistently, under extrapolation settings hardness shows an improvement tendency from MP to MPD/MPDD (Table 6 and Table 7).

In contrast, disintegration time depends not only on capillary infiltration and disintegrant swelling but also on latent states such as pore-network structure, wettability, and moisture history. As shown in Figure 14, features close to the disintegration mechanism (e.g., lipophilic score, loss on drying, and solubility score) vary substantially over time. In addition, the later-period formulation groups in this dataset included conditions with higher granule usage ratios and different proportions of some mineral-based raw materials, which may also have contributed to the observed covariate shift. Such changes can include mechanism switching (concept shift) due to process updates and material substitutions, which can lead to large errors that are not fully explained by distance-based applicability-domain (AD) indicators alone (Figure 12). As a result, disintegration time can degrade from MP to MPD/MPDD under extrapolation settings (Table 6 and Table 7), highlighting the need for descriptor selection and dimensionality (correlation) control, as well as AD-aware risk flagging in deployment. We therefore regard the reduced extrapolation performance for disintegration time as an important limitation of the present study. Expanding future datasets to cover a broader formulation space may reduce the frequency of true extrapolation in practical use, but this remains an important subject for future validation.

### 4.3. Physical Considerations

In this section, we qualitatively interpret trends in performance changes by considering the dominant mechanisms underlying each target variable and the extent to which the additional descriptors (MPD: feature set augmented with scalar descriptors; MPDD: MPD further augmented with PSD-derived summary features) can represent those mechanisms. The numbers in parentheses indicate the sample sizes used in the analysis.

Hardness (n=1209): Tablet strength is governed by plastic deformation/brittle fracture under compression and changes in interparticle contact and effective bonding area (packing state). Scalar descriptors such as density, hygroscopicity, and shape (MPD) correspond strongly to these average behaviors, making it reasonable that improvements relative to MP are observed even in metrics averaged across models. SHAP analysis also ranks compaction pressure highest and indicates contributions from category ratios, powder properties (e.g., Hausner ratio), and PSD summary features, consistent with the interpretation of dominant mechanisms. In particular, a higher main-component ratio can coincide with a lower relative fraction of excipients that promote compressibility or binding, which is consistent with a tendency toward lower hardness. Likewise, glidant-related features may reflect changes in powder packing and stress transmission before and during compaction. PSD information (MPDD) would in principle influence bonding area through fine-particle filling and bridging; however, high-dimensional and strongly correlated distribution features may reduce effect sizes. Nevertheless, improvements with MPDD are observed in both averaged metrics and best-model comparisons, suggesting that adding information related to bonding area can be beneficial.

Disintegration time (n=882): Disintegration is governed by capillary infiltration and swelling of disintegrants, with the pore-network structure and pore-size distribution being key factors. MPDD features such as mean particle size and wettability capture average infiltration behavior, and PSD summaries (MPDD) can serve as a proxy for pore-size distribution, increasing explanatory power. In the averaged metrics, both MPD and MPDD show additive benefits in the interpolation evaluation. In SHAP analysis, in addition to compaction pressure, “disintegrant ratio × granule ratio” and loss on drying rank highly, suggesting contributions from both composition/moisture state and compression conditions to disintegration behavior. Loss on drying may also reflect extract-related properties of raw materials, which can influence water uptake, swelling, and the resulting disintegration response.

Flow function (n=151): Flowability is sensitive to interparticle friction, shape anisotropy, fine-particle agglomeration, and small fluctuations in moisture, making it a metric for which experimental reproducibility can be difficult to ensure. Although MPD features related to shape and surface properties contribute, noise relative to signal may be large, resulting in small average effect sizes. The fine-particle-end information included in MPDD is useful, and improvements are observed in both averaged metrics and best-model comparisons. SHAP analysis also shows PSD summary features such as PSD fraction ≤ 25 μm and quantiles (PSD d10 (μm) and PSD d50 (μm)) among the top contributors, quantitatively supporting the contribution of particle-size design. This is physically plausible because an increased fine-particle fraction can increase cohesion and adhesion, thereby reducing flowability, whereas glidant-related features may contribute through mitigation of powder-surface interactions. At the same time, because the sample size is still limited relative to the feature dimensionality, these findings should be interpreted as exploratory rather than definitive.

Cohesion (n=145): Cohesion strongly depends on the fine-particle fraction, surface energy, and liquid bridging, and PSD is a primary determinant. However, in the interpolation evaluation of this study, the effect sizes themselves are small and improvements are limited even in averaged metrics. Under MPD, a slight degradation is observed on average, and additive benefits are not clear even for the best model. Under MPDD, neither significant improvement nor degradation is observed, leaving uncertainty in the additive benefit. In addition to the relatively small sample size (n=145) and large estimation variability, information relevant to cohesion (e.g., fine-particle-end and surface state) may not be sufficiently represented by distribution features alone. SHAP analysis ranks PSD fraction ≤25μm and PSD d50 (μm) highly, suggesting contributions of the fine end and representative particle size. An increase in the fine fraction is also physically consistent with stronger interparticle interactions and thus higher cohesion. However, because differences in importance among features are not large, interpretation should be made cautiously in light of uncertainty.

Thickness (n=60): Thickness is determined by fill density and compression response (elastic recovery), and PSD is in principle expected to be effective through void filling and packing. Consistently with this expectation, in the best-model comparison under random splitting, adding descriptors and PSD summaries yields a modest but monotonic reduction in RMSE from MP to MPD to MPDD (Table 5), suggesting that the additional features can be beneficial for thickness prediction. At the same time, when averaged across all models, the net effect is close to zero and can appear mixed (Table 4), indicating that gains are not universal across algorithms. Given the small sample size (n=60) and the limited degrees of freedom relative to high-dimensional features, the observed improvement should be interpreted as a promising but still uncertain signal that warrants confirmation with additional data. In SHAP analysis, compaction pressure and 1/Weight rank highly, suggesting that thickness depends strongly on geometry/scale and compression conditions. This is consistent with the direct geometric relationship between design weight and tablet thickness. The non-negligible contribution of formulation ratios such as glidant-related features may additionally reflect effects on pressure transmission, packing, and ejection behavior that influence the final post-compaction dimension.

### 4.4. Implications for Pre-Formulation Decision Making

Pre-formulation aims to reduce downstream development risk by screening candidate materials and compositions before extensive tablet prototyping. Because the proposed framework relies on raw-material properties, composition information, and PSD-derived summaries (MP/MPD/MPDD), it can be used as a virtual screening tool at the pre-formulation stage to rank candidate formulations and to prioritize confirmatory experiments under limited budgets. In particular, the SHAP-based interpretation suggests which material attributes and PSD characteristics (e.g., fine fraction and representative quantiles) are most influential for each target property, enabling formulation teams to translate model outputs into actionable design hypotheses (attribute windows) rather than treating predictions as black-box numbers. At the same time, the target properties considered here should not be interpreted independently. The physical considerations and SHAP trends suggest practical trade-offs: increasing the main-component ratio can reduce hardness, fine-particle and glidant-related features can improve powder handling but also alter cohesion and compaction behavior, and compression-related settings that strengthen tablets can also affect disintegration and final thickness. Accordingly, formulation design is inherently a multi-objective problem in which acceptable candidates must balance several competing requirements rather than optimize a single property. In this sense, the proposed framework is useful not only for single-property prediction but also for screening candidate regions that satisfy multiple property constraints simultaneously, and it may provide a basis for future inverse-design workflows.

For practical deployment, predictions should be conditioned on applicability-domain (AD) indicators, especially when evaluating formulation updates that induce covariate shift. The extrapolation-oriented results indicate that descriptor augmentation can improve hardness prediction while degrading disintegration-time prediction in some shifted regimes, highlighting the need for descriptor selection and dimensionality control. Therefore, we recommend using AD-aware screening to filter or down-weight low-coverage candidates and to trigger targeted additional measurements when predictions are requested outside the learned domain.

## 5. Conclusions

In this study, to support pre-formulation property prediction and formulation-design decision making in tablet development, we constructed feature sets that integrate materials and process information (MP) with scalar descriptors (D) and further with PSD information (DD), yielding MP/MPD/MPDD, and developed a suite of predictive models using machine learning and deep learning. For mixture systems, descriptors were formulated as composition-weighted sums. Reproducibility was assessed via Train/Dev/Test splitting and repeated experiments (n=50), and effects were evaluated using bootstrap CIs for the RMSE improvement *d* and Wilcoxon tests.

For Test results under interpolation prediction (Figure 3, Figure 4, Figure 5, Figure 6 and Figure 7), performance comparisons were provided, and consistent with the forest plot summarizing effect sizes (Figure 9), model-averaged mean improvements indicate that feature augmentation improves hardness and disintegration time on average and yields smaller but positive mean improvements for flow function, with larger gains for flow function under MPDD. In contrast, cohesion and thickness show limited net benefits when averaged across models, with mixed or near-zero mean changes.

The effect of descriptor augmentation was statistically supported by bootstrap CIs and Wilcoxon tests, and reproducibility was demonstrated by the directional-agreement rate across random splits, r=0.833 (k=50/n=60; 95% Clopper–Pearson CI: 0.715–0.917). By qualitatively organizing the correspondence between the additional features (descriptors and distribution features) and dominant mechanisms, we obtained insights into interpretability of model outputs.

For extrapolation evaluation, we employed rolling-origin time-series splitting to mimic practical temporal distribution shift between past (Train) and future (Test) data. As a result, for hardness, both MPD and MPDD yielded positive RMSE improvement *d* (lower RMSE), and $\Delta R^{2}$ and Spearman’s ρ were also positive, indicating an improvement tendency from adding descriptors and distribution features. In contrast, for disintegration time, $\Delta R^{2}$ and Δρ were negative and the RMSE improvement was also negative (higher RMSE), indicating an overall tendency toward degradation. In addition, Figure 12 suggests that the association between AD indicators and errors is generally weak and heterogeneous; for some indicators in some settings (including leverage for hardness), the upper envelope of errors shows only a modest increasing tendency at larger AD values. Thus, AD indicators are most useful as supplementary risk flags rather than as strong standalone predictors of error magnitude.

Overall, by integrating materials, process, descriptors, and PSD information and employing diverse learning models, we demonstrated that the predictive accuracy of tablet properties can be improved. In internal development settings, this framework has the potential to support shorter development timelines, reduced testing costs, and improved quality stability through more efficient exploration of formulation design and formalization of knowledge.

From a pre-formulation perspective, a practical workflow enabled by this study is: (i) measure a minimal set of raw-material properties and PSDs, (ii) construct MPD/MPDD features for candidate compositions, (iii) predict powder and tablet-relevant properties for early-stage ranking, and (iv) restrict decision making to candidates with sufficient AD coverage, followed by a small number of confirmatory prototypes. By turning raw-material measurements into quantitative, AD-aware predictions, the framework supports earlier go/no-go decisions and more efficient QbD-informed formulation exploration.

## Figures and Tables

**Table 1:** Examples of raw materials used in the formulations prepared in this study.

Category	Number of Materials Used	Examples
Diluents	8	Microcrystalline cellulose (MCC)
		Lactose
Disintegrants	9	Partially pregelatinized starch
		Sodium carboxymethyl cellulose
Polyols	4	Maltitol
		Erythritol
Lubricants	4	Magnesium stearate
		Calcium stearate
Binders	2	Hydroxypropyl methylcellulose (HPMC)
		Hydroxypropyl cellulose (HPC)
Glidants	2	Colloidal silicon dioxide

**Table 2:** Hyperparameter search ranges for machine learning models.

Model Name	Hyperparameter	Value
Extra Trees Regressor	Number of Estimators	50, 100
	Maximum Features	0.3, 0.5, 0.7
	Maximum Depth	None, 10, 20
	Minimum Samples per Leaf	1, 5, 10
	Minimum Samples for Split	2, 10
Random Forest Regressor	Number of Estimators	50, 100
	Maximum Features	0.3, 0.5, 0.7
	Maximum Depth	None, 10, 20
	Minimum Samples per Leaf	1, 2, 4
	Minimum Samples for Split	2, 5
SVR	Regularization Parameter (C)	0.001, 0.01, 0.1, 1, 10
	Kernel Function	linear, rbf
	Kernel Coefficient (γ)	0.1, 0.5, 1.0
PLS	Number of Components	1 to 10
Lasso	Regularization Parameter (α)	0.001, 0.01, 0.1, 1, 10

**Table 3:** Hyperparameter search ranges for the deep learning model.

Parameter Category	Parameter	Value
Basic Settings	Learning Rate	$1.0\times 10^{-5}$ to $1.0\times 10^{-2}$
	Activation Function	ReLU, Tanh, ELU, Swish
	Dropout Rate	0.1 to 0.5
Descriptor Network	Number of Layers	1 to 3
	Hidden Layer Units	10 to 190
Distribution Network	Number of Layers	1 to 3
	Hidden Layer Units	10 to 190
Concatenated Network	Number of Layers	1 to 3
	Hidden Layer Units	10 to 190
Training Settings	Batch Size	8, 16, 32
	Number of Epochs	50 to 300

**Table 4:** Model-averaged performance improvements from MP to MPD/MPDD under interpolation settings (Test; mean over 6 models × 50 random splits, target-wise).

Target	Metric	Baseline (MP)	Improvement (MP → MPD)	Improvement (MP → MPDD)
Hardness	$R^{2}$	0.768	0.040↑	0.039↑
	RMSE	21.611	1.733↑	1.631↑
Disintegration time	$R^{2}$	0.657	0.059↑	0.062↑
	RMSE	8.874	0.719↑	0.768↑
Flow function	$R^{2}$	0.591	0.029↑	0.064↑
	RMSE	2.141	0.057↑	0.152↑
Cohesion	$R^{2}$	0.635	−0.047↓	0.013↑
	RMSE	0.131	−0.008↓	0.002↑
Thickness	$R^{2}$	0.737	−0.041↓	−0.041↓
	RMSE	0.129	−0.002↓	−0.006↓

Note: Improvements are defined as $R^{2}\left(\mathrm{MPD}/\mathrm{MPDD}\right)-R^{2}\left(\mathrm{MP}\right)$ for $R^{2}$ and RMSE(MP)−RMSE(MPD/MPDD) for RMSE, so that positive values consistently indicate improvement. Arrows in the table indicate interpretation of improvement values; ↑ denotes improvement and ↓ denotes degradation. Baseline (MP) performance indicates the mean performance under MP across all models for each target variable. Improvements indicate the mean across all trials of the six learning models × repeated random splits (*n* = 50).

**Table 5:** Performance comparison of the best models under interpolation settings (Test; target-wise mean).

Target	Metric	MP	MPD	MPDD
Hardness	$R^{2}$	0.887	0.901	0.899
	RMSE	15.596	14.577	14.781
Disintegration time	$R^{2}$	0.809	0.819	0.801
	RMSE	6.661	6.487	6.796
Flow function	$R^{2}$	0.687	0.700	0.700
	RMSE	1.913	1.861	1.868
Cohesion	$R^{2}$	0.726	0.741	0.733
	RMSE	0.116	0.113	0.114
Thickness	$R^{2}$	0.980	0.983	0.983
	RMSE	0.044	0.039	0.038

**Table 6:** Summary under extrapolation settings (time-series CV) for Hardness and Disintegration time (aggregated across multiple models): baseline performance (MP) and mean performance improvements (five models excluding NN).

**Baseline (MP) performance and improvements for Hardness**			
**Metric**	**Baseline (MP)**	**Improvement (MP → MPD)**	**Improvement (MP → MPDD)**
$R^{2}$	−0.193	0.225↑	0.232↑
RMSE	20.4	1.8↑	1.6↑
ρ	0.663	0.037↑	0.027↑
**Baseline (MP) performance and improvements for Disintegration time**			
**Metric**	**Baseline (MP)**	**Improvement (MP → MPD)**	**Improvement (MP → MPDD)**
$R^{2}$	0.102	−0.684↓	−0.693↓
RMSE	9.489	−1.471↓	−1.695↓
ρ	0.632	−0.074↓	−0.190↓

Note: The arrows indicate interpretation of the (mean) improvement; ↑ denotes improvement and ↓ denotes degradation. Improvements were computed as $R^{2}\left(\mathrm{MPD}/\mathrm{MPDD}\right)-R^{2}\left(\mathrm{MP}\right)$ and ρ(MPD/MPDD)−ρ(MP) for $R^{2}$ and ρ, and RMSE(MP)−RMSE(MPD/MPDD) for RMSE, so that positive values consistently indicate improvement.

**Table 7:** Additional supplementary evaluation under extrapolation settings (time-series CV) for Hardness and Disintegration time: comparison of metrics with the best MP model fixed (Test average).

**Performance comparison for the highest-performing model for Hardness (RF)**			
Metric	**Performance (MP)**	**Performance (MPD)**	**Performance (MPDD)**
$R^{2}$	0.380	0.521	0.505
RMSE (N)	15.902	13.965	14.271
ρ	0.752	0.809	0.814
**Performance comparison for the highest-performing model for Disintegration time (Lasso)**			
Metric	**Performance (MP)**	**Performance (MPD)**	**Performance (MPDD)**
$R^{2}$	0.408	−2.335	−2.031
RMSE (minute)	8.006	14.787	14.178
ρ	0.718	0.564	0.551

**Table 8:** AD coverage under rolling-origin time-series CV (fold-averaged, Train+Dev/Test).

Target	Pattern	Split	Lev (%)	MD (%)	kNN (%)	Range OK (%)
Hardness	MP	Train+Dev	94.9	92.2	97.3	100.0
		Test	43.8	100.0	81.5	99.7
	MPD	Train+Dev	94.9	87.3	97.3	100.0
		Test	23.1	93.6	75.2	99.7
	MPDD	Train+Dev	94.9	86.2	97.1	100.0
		Test	21.8	93.9	75.5	99.7
Disintegration time	MP	Train+Dev	95.0	96.3	97.6	100.0
		Test	67.5	100.0	86.1	99.8
	MPD	Train+Dev	95.0	89.1	96.3	100.0
		Test	40.2	92.3	80.9	99.7
	MPDD	Train+Dev	95.0	88.5	97.1	100.0
		Test	37.5	92.7	80.9	99.7

## Data Availability

To support transparency and reproducibility, the Supporting Information provides a detailed description of the preprocessing workflow, feature-set construction (MP/MPD/MPDD), data-splitting design, hyperparameter search ranges, and statistical evaluation procedures, together with representative anonymized sample data. Detailed formulation data cannot be made publicly available due to our continued use in proprietary product development. However, these data can be made available from the corresponding author upon reasonable request (M. Hamaguchi, hamaguchi@keio.jp), subject to confidentiality considerations.

## Supplementary Materials

The following supporting information can be downloaded at: https://www.mdpi.com/article/10.3390/pharmaceutics18040452/s1. The Supporting Information summarizes the preprocessing workflow, feature-set construction (MP/MPD/MPDD), data-splitting design, and statistical evaluation methods. It also provides dataset-structure and descriptor tables (Tables S1 and S2), detailed hyperparameter-search ranges (Tables S3 and S4), representative anonymized sample data (Tables S5–S7), full random- and time-split result tables (Tables S8–S17), representative parity plots (Figures S1–S5), additional SHAP plots (Figures S6–S8), and supplementary covariate-shift summaries (Figures S9–S14 and Table S18).
