# Supplementary material for: Application of AI in Tablet Development: An Integrated Machine Learning Framework for Pre-Formulation Property Prediction
Source: Pharmaceutics. 2026 Apr 8;18(4):452. doi: 10.3390/pharmaceutics18040452 (PMC13118576; doi:10.3390/pharmaceutics18040452)
Supplement: Supplementary file 1 [file pharmaceutics-18-00452-s001.zip › pharmaceutics-4200764-Supplementary.pdf]

# Supplementary Materials: Application of AI in Tablet Development: An Integrated Machine Learning Framework for Pre-formulation Property Prediction: Supporting Information

**Masugu Hamaguchi[1,2,*], Tomoki Adachi[3] and Noriyoshi Arai[1]**

## Supporting Information

This Supporting Information expands the *Data Availability Statement* in the main manuscript. It provides a structured location to disclose the preprocessing workflow, data-splitting design, hyperparameter search ranges, and statistical methods, and to describe aggregated statistics and analysis outputs. An anonymized representative sample dataset is provided in this Supporting Information to facilitate reproducibility checks. Full formulation records and the full source code are available from the corresponding author upon reasonable request, subject to confidentiality considerations.

## S1. Preprocessing Flow (Validation → Conversion → Storage)

### *S1.1. Data Sources and Unified Schema*

The evaluation workflow assumes that each record can be represented in a unified tabular schema suitable for supervised learning. The implementation details of data storage and retrieval are out of scope for the Supporting Information; here we describe only the schema required for AI model construction.

The unified schema used by the evaluation services consists of:

- **Material-composition features**: formulation composition by material ID, aligned to the full materials master and missing values filled with 0.
- **Process-condition features**: process and design variables used in tableting (e.g., compaction force, punch type, tablet diameter, etc.).
- **Target properties**: Hardness, Disintegration Time, Flow function, Cohesion, and Thickness.

### *S1.2. Dataset Structure by Target Variable*

To clarify dataset structure, we audited each target variable using unique formulations, defined on a material-composition basis. For Hardness, Disintegration Time, and Thickness, we additionally report unique conditions, defined on a combined material-composition and process-condition basis. For Flow function and Cohesion, which are pre-compression powder properties, dataset structure is summarized on a material-composition basis only.

**Table S1.** Dataset structure by target variable. Unique formulations are defined on a material-composition basis. Unique conditions are additionally reported for Hardness, Disintegration Time, and Thickness on a combined material-composition and process-condition basis. For Flow function and Cohesion, the condition-based criterion was not adopted.

| Target variable | $n_{\text{samples}}$ | $n_{\text{unique formulations}}$ | $n_{\text{unique conditions}}$ | Duplicate conditions | Duplicate-condition rate |
|---|---|---|---|---|---|
| Hardness | 1209 | 388 | 998 | 211 | 0.175 |
| Disintegration Time | 882 | 371 | 698 | 184 | 0.209 |
| Flow function | 151 | 136 | N/A | N/A | N/A |
| Cohesion | 145 | 134 | N/A | N/A | N/A |
| Thickness | 60 | 23 | 60 | 0 | 0.000 |

This audit indicates that Hardness and Disintegration Time include multiple observations that share identical formulation compositions and the same recorded process-

condition variables. In contrast, Thickness includes repeated formulations but no repeated formulation–process conditions. For Flow function and Cohesion, the dataset structure is summarized on a material-composition basis only; under this definition, the counts were 136 unique formulations among 151 observations and 134 unique formulations among 145 observations, respectively. Accordingly, the dataset is not fully independent for all targets.

### *S1.3. Feature Construction Summary*

Three feature sets were constructed to evaluate the contribution of descriptor information:

- **MP** (Materials + Processes): a 157-variable (base) feature set combining material-composition features and process-condition features.
- **MPD** (MP + scalar Descriptors): MP augmented with a scalar-descriptor block. The scalar-descriptor dimensionality is target-specific (37–45 dimensions in this study; see Table S2).
- **MPDD** (MPD + particle size Distribution): MPD further augmented with 10-dimensional summary features derived from an 80-bin mixture particle size distribution.

**Breakdown of MP (157 base variables)**: the MP block is defined before one-hot encoding as

$$157 \;=\; N_{\mathrm{mat}} + N_{\mathrm{proc,cont}} + N_{\mathrm{proc,cat}},$$

where $N_{\mathrm{mat}} = 151$ material-composition variables (one column per material ID in the materials master, aligned and zero-filled), $N_{\mathrm{proc,cont}} = 5$ continuous process variables ({compaction force, tablet diameter, tablet weight, curvature radius (1-layer), curvature radius (2-layer)}), and $N_{\mathrm{proc,cat}} = 1$ categorical process variable (punch type). In preprocessing, the categorical punch type is one-hot encoded (yielding $K$ dummy variables; in our dataset $K = 2$), so the numeric input to models replaces the single punch-type column with $K$ one-hot columns while keeping all other MP variables unchanged (thus the numeric MP input has $151 + 5 + 2 = 158$ dimensions in our dataset).

**Scalar descriptor features**: powder-property values are aggregated to mixture-level descriptors by a composition-weighted average. In addition to powder-property values, derived scalars based on mixing and design are included. Missing values for scalar descriptors are imputed using the $k$-nearest neighbors method ($k = 5$). A target-specific subset of scalar-descriptor columns is predefined in the evaluation configuration.

#### S1.3.1. Scalar Descriptor Dimensions (Target-Specific)

The scalar descriptor pool used in MPD/MPDD is defined by the descriptor-construction procedure described above. In practice, a target-specific subset of scalar descriptors is selected a priori for each target property; Table S2 summarizes which scalar descriptors are used for each target. The scalar descriptor block used in this study consists of:

- **Target-specific scalar material-property descriptors and derived scores**: mixture-level descriptors obtained by composition-weighted aggregation of raw-material properties (e.g., bulk densities, compressibility, Hausner ratio, loss on drying, FLODEX/flow-function-related measures, and derived expert scores such as a 3-level flowability score (promoting/neutral/inhibiting), disintegration tendency, solubility, and lipophilicity).
- **Engineered scalar descriptors**: derived scalars based on tablet geometry and process conditions (e.g., curvature-related quantities, packing-state proxies, inverse tablet weight, and compaction pressure).

- **9 material-group ratio descriptors** computed from formulation compositions (group-wise sums divided by tablet weight) and **21 second-order interaction terms** generated from 7 ratio descriptors (microcrystalline cellulose through granule; pairwise product interactions; active component and other fraction are excluded from the interaction generator; squared terms are not included).

The resulting scalar-descriptor dimensionality (including ratios and interactions) was: Hardness=44, Disintegration Time=45, Flow function=37, Cohesion=37, and Thickness=43.

**Table S2.** Target-specific scalar descriptor selection. A checkmark indicates that the descriptor (or descriptor block) is used for the corresponding target.

| Descriptor | Hardness | Disintegration Time | Flow function | Cohesion | Thickness |
|---|---|---|---|---|---|
| Loose bulk density | ✓ | ✓ | ✓ | ✓ | ✓ |
| Tapped bulk density | ✓ | ✓ | ✓ | ✓ | ✓ |
| Compressibility (Carr's index) | ✓ | ✓ | ✓ | ✓ | ✓ |
| Hausner ratio | ✓ | ✓ | ✓ | ✓ | ✓ |
| FLODEX | | | ✓ | ✓ | |
| Loss on drying | ✓ | ✓ | ✓ | ✓ | ✓ |
| Solubility score | | ✓ | | | |
| Lipophilic score | | ✓ | | | |
| Flowability score | | | ✓ | ✓ | |
| R-part height (engineered) | ✓ | ✓ | | | ✓ |
| R-part volume (engineered) | ✓ | ✓ | | | ✓ |
| Inverse tablet weight (engineered) | ✓ | ✓ | | | ✓ |
| compaction pressure (engineered) | ✓ | ✓ | | | ✓ |
| Microcrystalline cellulose fraction | ✓ | ✓ | ✓ | ✓ | ✓ |
| Disintegrant fraction | ✓ | ✓ | ✓ | ✓ | ✓ |
| Sugar alcohol fraction | ✓ | ✓ | ✓ | ✓ | ✓ |
| Lubricant fraction | ✓ | ✓ | ✓ | ✓ | ✓ |
| Binder fraction | ✓ | ✓ | ✓ | ✓ | ✓ |
| Glidant fraction | ✓ | ✓ | ✓ | ✓ | ✓ |
| Granule fraction | ✓ | ✓ | ✓ | ✓ | ✓ |
| Active component fraction | ✓ | ✓ | ✓ | ✓ | ✓ |
| Other fraction | ✓ | ✓ | ✓ | ✓ | ✓ |
| 21 second-order interaction terms from 7 fractions (microcrystalline cellulose through granule) | ✓ | ✓ | ✓ | ✓ | ✓ |

**Particle size distribution features**: particle size distribution (PSD) information is originally available as an 80-bin frequency-distribution vector per raw material, spanning 0.115 $\mu$m to 5000 $\mu$m. A prescription-level mixture PSD is constructed by a composition-weighted aggregation of the raw-material PSDs; when a raw material's PSD is unavailable, it is treated as an all-zero vector (i.e., it contributes no PSD information to the mixture). The resulting mixture PSD is then row-normalized to sum to 1.

Because the 80-bin PSD is high-dimensional relative to the sample size for several targets, we do not use the full PSD vector directly as a model input. Instead, we extract a low-dimensional set of distribution-summary features from the normalized mixture PSD. Specifically, we compute: (i) percentile particle sizes $d_{10}$, $d_{50}$, and $d_{90}$ (obtained by interpolating the cumulative distribution function); (ii) mean and standard deviation of particle size; (iii) skewness and (excess) kurtosis; (iv) tail fractions (fraction $\leq 25\,\mu$m and fraction $\geq 150\,\mu$m); and (v) Shannon entropy of the mixture PSD. These 10 summary features constitute the PSD block in MPDD.

**Feature-set structure**: MP comprises materials and process features. MPD augments MP with the target-specific scalar-descriptor block (37–45 dimensions in this study). MPDD further augments MPD with the 10-dimensional PSD summary feature block derived from the mixture PSD. For deep learning, these three blocks are input as separate branches and concatenated after block-specific processing.

**Standardization / encoding**: standardization to mean 0 and standard deviation 1 is applied to continuous explanatory variables except formulation composition (material amounts / fractions), which are not standardized. Scalar descriptors and the PSD summary features are standardized in the same way as other continuous numeric variables. Categorical process variables (e.g., punch type) are one-hot encoded within the preprocessing pipeline.

## S2. Data Splitting Design

### *S2.1. In-Distribution Evaluation (Random Split): Train/Dev/Test (5:3:2) and Iterative Seeds*

For both neural-network and machine-learning evaluation scripts, an in-distribution evaluation with a random split is used:

- **Test split**: 20% held out as Test with shuffling; the split is determined by the trial random seed.
- **Train/Dev split**: the remaining 80% is split into Train and Dev such that Dev=30% of total and Train=50% of total, using the same trial random seed.

Model training follows a **Train-only training / Train+Dev retraining** scheme:

- Hyperparameters are selected using Train/Dev (Dev acts as the validation set).
- After selecting hyperparameters, a model is retrained on Train+Dev and evaluated on Test.

To mitigate dependence on split random seeds, iterative experiments were conducted with varying seeds ($n = 50$). Each seed defines a complete Train/Dev/Test split, and performance is summarized by averaging across trials.

### *S2.2. Extrapolation-Oriented Evaluation: Rolling-Origin Time-Series Splitting*

For extrapolation-oriented evaluation, a rolling-origin time-series splitting procedure is implemented. The sample index order follows the chronological measurement order (oldest to newest); no shuffling is applied.

- **Folds**: $K = 5$ folds are generated over the sample index order.
- **Per-fold windows**: Train=50%, Dev=20%, Test=10% (non-overlapping contiguous blocks).
- **Step size**: the train end is advanced by 5% per fold (computed as the remaining proportion divided by $K - 1$).
- Folds that cannot fit full Dev/Test windows are skipped to avoid overlap and incomplete windows.

### *S2.3. Applicability Domain (AD) Indicators*

Applicability Domain (AD) coverage is computed with thresholds fit on the Train+Dev set (the same data used for final model retraining). The following indicators are implemented:

- **Leverage**: hat-matrix leverage $h(x)$ computed via a pseudo-inverse of $(X^\top X)$. Threshold is the 95th percentile of the Train+Dev leverage distribution by default (optionally $3p/n$ is supported but disabled by default).
- **Mahalanobis distance**: features are centered; PCA is used to retain 95% variance, and covariance is estimated using Ledoit–Wolf shrinkage by default. The threshold is a $\chi^2$ quantile with $\alpha = 0.05$ (threshold is defined in squared-distance space).
- **kNN mean distance**: for each sample, the mean distance to the $k = 5$ nearest neighbors in Train+Dev. Threshold is the 95th percentile of the Train+Dev kNN-mean distribution.
- **Range check**: per-feature Train+Dev min/max bounds are used; the percentage of samples within the allowed range (tolerance=0% by default) is reported.

## S3. Hyperparameter Search Ranges

### *S3.1. Machine Learning Models*

Machine-learning models are tuned by grid search within each random-split trial, with the objective of minimizing mean squared error (MSE) on Dev.

**Table S3.** Hyperparameter search ranges for machine learning models (grid search).

| Model | Hyperparameter | Range |
|---|---|---|
| ET | n_estimators | 50, 100 |
| ET | max_features | 0.3, 0.5, 0.7 |
| ET | max_depth | None, 10, 20 |
| ET | min_samples_leaf | 1, 5, 10 |
| ET | min_samples_split | 2, 10 |
| RF | n_estimators | 50, 100 |
| RF | max_features | 0.3, 0.5, 0.7 |
| RF | max_depth | None, 10, 20 |
| RF | min_samples_leaf | 1, 2, 4 |
| RF | min_samples_split | 2, 5 |
| SVR | C | 0.001, 0.01, 0.1, 1, 10 |
| SVR | kernel | linear, rbf |
| SVR | gamma | 0.1, 0.5, 1.0 |
| Lasso | alpha | 0.001, 0.01, 0.1, 1, 10 |
| PLS | n_components | 1–10 |

ET: Extra Trees Regressor; RF: Random Forest Regressor; SVR: Support Vector Regression; PLS: Partial Least Squares.

### *S3.2. Deep Learning Model*

The neural-network model is tuned by Bayesian optimization (Optuna) with a Train/Dev split and an objective of minimizing Dev RMSE. Early stopping and learning-rate decay are applied during training.

**Table S4.** Hyperparameter search ranges for the deep learning model (Optuna).

| Hyperparameter | Range |
|---|---|
| learning_rate | $10^{-5}$ to $10^{-2}$ (log-uniform) |
| activation | relu, tanh, elu, swish |
| dropout_rate | 0.1 to 0.5 |
| n_layer_deep_desc | 1 to 3 |
| deep_mid_units_desc | 10 to 190 (step 20) |
| n_layer_deep_distribution | 1 to 3 |
| deep_mid_units_distribution | 10 to 190 (step 20) |
| n_layer_shallow | 1 to 3 |
| shallow_mid_units | 10 to 190 (step 20) |
| batch_size | 8, 16, 32 |
| epochs | 50 to 300 (step 10) |

The number of Optuna trials was 50 per configuration while confirming convergence.

## S4. Statistical Methods for Effect Verification

### *S4.1. RMSE Improvement Definition*

Under identical conditions (same learning model and data split), baseline (MP) and expanded feature sets ($F \in \{\text{MPD}, \text{MPDD}\}$) were paired, and the RMSE improvement was defined as:

$$d = \text{RMSE}(\text{MP}) - \text{RMSE}(F).$$

Here, $d > 0$ indicates improved predictive accuracy (RMSE reduction).

### *S4.2. Bootstrap Confidence Intervals*

For each feature set, the mean improvement $\bar{d}$ by learning model and its 95% bootstrap confidence interval were computed using the percentile method with $B = 50{,}000$ resamples.

*S4.3. Paired Wilcoxon Signed-Rank Tests*

Two-sided paired Wilcoxon signed-rank tests were performed on $\{d_i\}$ with significance threshold $p < 0.05$. Model-wise multiple comparisons were treated as exploratory. Accordingly, $p$-values are reported without multiplicity correction; effect sizes and confidence intervals are emphasized.

*S4.4. Directional Reproducibility across Discovery/Validation Seed Sets*

For reproducibility, random seeds were split into non-overlapping discovery and validation sets. Directional concordance ($\bar{d} > 0$ in both sets) and its exact 95% binomial confidence interval (Clopper–Pearson) were reported.

## S5. Sample Data

An anonymized representative sample dataset is provided as part of this Supporting Information. The sample data consists of a representative subset with anonymized material identifiers; values are scaled/rounded as needed to reduce re-identification risk. Included variables are formulation inputs (raw materials and process variables) and response variables (tablet property targets), where available. Some response values are intentionally left blank in the sample dataset.

**Table S5.** Representative anonymized sample prescriptions, part (a). Units of all Material columns are (mg).

| Sample ID | Disintegration time (min) | Thickness (mm) | Hardness (N) | Flow function | Cohesion | Tablet diameter (mm) | Curvature radius (mm) | Compression load (kgf) | Tablet weight (mg) | Punch type | Material A0005 | Material A0008 | Material B0002 | Material B0003 | Material B0005 | Material B0006 | Material C0001 | Material C0005 |
|---|---|---|---|---|---|---|---|---|---|---|---|---|---|---|---|---|---|---|
| 1 | 55.7 | 4.2 | 150.765 | N/A | N/A | 8 | 10 | 500 | 250 | Standard R | 42.7893 | 0 | 7.5 | 0 | 0 | 0 | 0 | 0 |
| 2 | 65.64 | 4.198 | 153.313 | N/A | N/A | 8 | 10 | 800 | 250 | Standard R | 42.7893 | 0 | 7.5 | 0 | 0 | 0 | 0 | 0 |
| 3 | 65.63 | 4.176 | 157.696 | 10.1196 | 0.318 | 8 | 10 | 1000 | 250 | Standard R | 42.7893 | 0 | 7.5 | 0 | 0 | 0 | 0 | 0 |
| 4 | 61.29 | 4.274 | 144.750 | 6.81859 | 0.506 | 8 | 10 | 1300 | 250 | Standard R | 42.7893 | 0 | 7.5 | 0 | 0 | 0 | 0 | 0 |
| 5 | 66.53 | 4.23 | 148.828 | 5.44271 | 0.625 | 8 | 10 | 1600 | 250 | Standard R | 42.7893 | 0 | 7.5 | 0 | 0 | 0 | 0 | 0 |
| 6 | 73.94 | 4.194 | 154.536 | 3.30294 | 0.964 | 8 | 10 | 2000 | 250 | Standard R | 42.7893 | 0 | 7.5 | 0 | 0 | 0 | 0 | 0 |
| 7 | 44.57 | 4.262 | 128.033 | 8.08795 | 0.412 | 8 | 10 | 500 | 250 | Standard R | 36.7893 | 12.5 | 5 | 0 | 0 | 0 | 0 | 0 |
| 8 | 50.33 | 4.21 | 132.722 | 9.44707 | 0.349 | 8 | 10 | 1000 | 250 | Standard R | 36.7893 | 12.5 | 5 | 0 | 0 | 0 | 0 | 0 |
| 9 | 51.73 | 4.194 | 133.028 | 6.69222 | 0.489 | 8 | 10 | 1300 | 250 | Standard R | 36.7893 | 12.5 | 5 | 0 | 0 | 0 | 0 | 0 |
| 10 | 48.72 | 4.274 | 125.382 | 5.71743 | 0.609 | 8 | 10 | 1600 | 250 | Standard R | 36.7893 | 12.5 | 5 | 0 | 0 | 0 | 0 | 0 |
| 11 | 53.54 | 4.242 | 131.193 | 3.4292 | 0.902 | 8 | 10 | 2000 | 250 | Standard R | 36.7893 | 12.5 | 5 | 0 | 0 | 0 | 0 | 0 |
| 12 | 56.13 | 4.19 | 136.697 | 6.76663 | 0.51 | 8 | 10 | 500 | 250 | Standard R | 20.2893 | 12.5 | 2.5 | 0 | 0 | 0 | 0 | 12.5 |
| 13 | N/A | N/A | N/A | 5.6214 | 0.592 | 8 | 6.5 | 1000 | 260 | Standard R | 59.8262 | 0 | 5.2 | 0 | 5.2 | 0 | 16.38 | 0 |
| 14 | N/A | N/A | N/A | 5.39556 | 0.582 | 8 | 6.5 | 1000 | 260 | Standard R | 59.8262 | 0 | 0 | 5.2 | 5.2 | 0 | 16.38 | 0 |
| 15 | N/A | N/A | N/A | 5.29099 | 0.63 | 8 | 6.5 | 1000 | 260 | Standard R | 65.0262 | 0 | 0 | 0 | 5.2 | 0 | 16.38 | 0 |
| 16 | N/A | N/A | N/A | 9.05687 | 0.37 | 8 | 6.5 | 1000 | 260 | Standard R | 59.8262 | 0 | 0 | 0 | 5.2 | 5.2 | 16.38 | 0 |
| 17 | N/A | N/A | 83.282 | 8.78098 | 0.385 | 8 | 6.5 | 700 | 250 | Standard R | 53.975 | 0 | 12.5 | 0 | 0 | 5 | 12.5 | 0 |
| 18 | N/A | N/A | 86.748 | N/A | N/A | 8 | 6.5 | 700 | 250 | Standard R | 53.975 | 0 | 12.5 | 0 | 0 | 5 | 12.5 | 0 |
| 19 | N/A | N/A | 78.899 | N/A | N/A | 8 | 6.5 | 700 | 250 | Standard R | 53.975 | 0 | 12.5 | 0 | 0 | 5 | 12.5 | 0 |

**Table S6.** Representative anonymized sample prescriptions, part (b). Units of all Material columns are (mg).

| Sample ID | Material D0001 | Material D0002 | Material E0001 | Material E0002 | Material F0001 | Material F0002 | Material G0004 | Material H0001 | Material H0004 | Material I0001 | Material I0003 | Material I0004 | Material I0005 |
|---|---|---|---|---|---|---|---|---|---|---|---|---|---|
| 1 | 0 | 3.75 | 0 | 0 | 2.5 | 5 | 12.5 | 152.011 | 0 | 0 | 0 | 4.3 | 0 |
| 2 | 0 | 3.75 | 0 | 0 | 2.5 | 5 | 12.5 | 152.011 | 0 | 0 | 0 | 4.3 | 0 |
| 3 | 0 | 3.75 | 0 | 0 | 2.5 | 5 | 12.5 | 152.011 | 0 | 0 | 0 | 4.3 | 0 |
| 4 | 0 | 3.75 | 0 | 0 | 2.5 | 5 | 12.5 | 152.011 | 0 | 0 | 0 | 4.3 | 0 |
| 5 | 0 | 3.75 | 0 | 0 | 2.5 | 5 | 12.5 | 152.011 | 0 | 0 | 0 | 4.3 | 0 |
| 6 | 0 | 3.75 | 0 | 0 | 2.5 | 5 | 12.5 | 152.011 | 0 | 0 | 0 | 4.3 | 0 |
| 7 | 0 | 3.75 | 0 | 1 | 2.5 | 5 | 7.5 | 152.011 | 0 | 0 | 0 | 4.3 | 0 |
| 8 | 0 | 3.75 | 0 | 1 | 2.5 | 5 | 7.5 | 152.011 | 0 | 0 | 0 | 4.3 | 0 |
| 9 | 0 | 3.75 | 0 | 1 | 2.5 | 5 | 7.5 | 152.011 | 0 | 0 | 0 | 4.3 | 0 |
| 10 | 0 | 3.75 | 0 | 1 | 2.5 | 5 | 7.5 | 152.011 | 0 | 0 | 0 | 4.3 | 0 |
| 11 | 0 | 3.75 | 0 | 1 | 2.5 | 5 | 7.5 | 152.011 | 0 | 0 | 0 | 4.3 | 0 |
| 12 | 0 | 3.75 | 0 | 7.5 | 2.5 | 5 | 7.5 | 152.011 | 0 | 0 | 0 | 4.3 | 0 |
| 13 | 2.6 | 0 | 2.6 | 0 | 2.6 | 0 | 0 | 0 | 69.9583 | 3.11112 | 4.65513 | 0 | 0.213807 |
| 14 | 2.6 | 0 | 2.6 | 0 | 2.6 | 0 | 0 | 0 | 69.9583 | 3.11112 | 4.65513 | 0 | 0.213807 |
| 15 | 2.6 | 0 | 2.6 | 0 | 2.6 | 0 | 0 | 0 | 69.9583 | 3.11112 | 4.65513 | 0 | 0.213807 |
| 16 | 2.6 | 0 | 2.6 | 0 | 2.6 | 0 | 0 | 0 | 69.9583 | 3.11112 | 4.65513 | 0 | 0.213807 |
| 17 | 2.5 | 0 | 0 | 0 | 0 | 0 | 0 | 0 | 69.95 | 3.125 | 4.675 | 0 | 0.2 |
| 18 | 2.5 | 0 | 0 | 0 | 0 | 0 | 0 | 0 | 69.95 | 3.125 | 4.675 | 0 | 0.2 |
| 19 | 2.5 | 0 | 0 | 0 | 0 | 0 | 0 | 0 | 69.95 | 3.125 | 4.675 | 0 | 0.2 |

**Table S7.** Representative anonymized sample prescriptions, part (c). Units of all Material columns are (mg).

| Sample ID | Material I0006 | Material I0007 | Material I0011 | Material I0017 | Material I0018 | Material I0020 | Material I0022 | Material I0024 | Material J0002 | Material J0009 | Material J0012 | Material K0013 | Material K0014 |
|---|---|---|---|---|---|---|---|---|---|---|---|---|---|
| 1 | 0 | 2.65 | 3.125 | 0 | 0 | 0 | 0 | 0 | 0 | 0 | 0 | 3.5 | 10.375 |
| 2 | 0 | 2.65 | 3.125 | 0 | 0 | 0 | 0 | 0 | 0 | 0 | 0 | 3.5 | 10.375 |
| 3 | 0 | 2.65 | 3.125 | 0 | 0 | 0 | 0 | 0 | 0 | 0 | 0 | 3.5 | 10.375 |
| 4 | 0 | 2.65 | 3.125 | 0 | 0 | 0 | 0 | 0 | 0 | 0 | 0 | 3.5 | 10.375 |
| 5 | 0 | 2.65 | 3.125 | 0 | 0 | 0 | 0 | 0 | 0 | 0 | 0 | 3.5 | 10.375 |
| 6 | 0 | 2.65 | 3.125 | 0 | 0 | 0 | 0 | 0 | 0 | 0 | 0 | 3.5 | 10.375 |
| 7 | 0 | 2.65 | 3.125 | 0 | 0 | 0 | 0 | 0 | 0 | 0 | 0 | 3.5 | 10.375 |
| 8 | 0 | 2.65 | 3.125 | 0 | 0 | 0 | 0 | 0 | 0 | 0 | 0 | 3.5 | 10.375 |
| 9 | 0 | 2.65 | 3.125 | 0 | 0 | 0 | 0 | 0 | 0 | 0 | 0 | 3.5 | 10.375 |
| 10 | 0 | 2.65 | 3.125 | 0 | 0 | 0 | 0 | 0 | 0 | 0 | 0 | 3.5 | 10.375 |
| 11 | 0 | 2.65 | 3.125 | 0 | 0 | 0 | 0 | 0 | 0 | 0 | 0 | 3.5 | 10.375 |
| 12 | 0 | 2.65 | 3.125 | 0 | 0 | 0 | 0 | 0 | 0 | 0 | 0 | 3.5 | 10.375 |
| 13 | 1.92426 | 0 | 2.15278 | 0 | 10.521 | 38.2605 | 2.4 | 2.1978 | 19.22 | 1.64578 | 9.33333 | 0 | 0 |
| 14 | 1.92426 | 0 | 2.15278 | 0 | 10.521 | 38.2605 | 2.4 | 2.1978 | 19.22 | 1.64578 | 9.33333 | 0 | 0 |
| 15 | 1.92426 | 0 | 2.15278 | 0 | 10.521 | 38.2605 | 2.4 | 2.1978 | 19.22 | 1.64578 | 9.33333 | 0 | 0 |
| 16 | 1.92426 | 0 | 2.15278 | 0 | 10.521 | 38.2605 | 2.4 | 2.1978 | 19.22 | 1.64578 | 9.33333 | 0 | 0 |
| 17 | 1.95 | 0 | 2.175 | 1.225 | 10.525 | 45.625 | 0.7 | 2.2 | 19.225 | 1.95 | 0 | 0 | 0 |
| 18 | 1.95 | 0 | 2.175 | 1.225 | 10.525 | 45.625 | 0.7 | 2.2 | 19.225 | 1.95 | 0 | 0 | 0 |
| 19 | 1.95 | 0 | 2.175 | 1.225 | 10.525 | 45.625 | 0.7 | 2.2 | 19.225 | 1.95 | 0 | 0 | 0 |

# S6. Evaluation Results

## *S6.1. Random-Split Evaluation Results (All Data)*

This section summarizes the random-split evaluation results aggregated over repeated trials. For each target property, we report Test RMSE and Test $R^2$.

**Table S8.** Random-split results (all data): Hardness. Mean Test RMSE and Test $R^2$ over 50 repeated random-split trials are shown for each model and feature set (MP, materials + processes; MPD, MP + scalar descriptors; MPDD, MPD + PSD summary features).

| Model | Feature set | Test RMSE (N) | Test $R^2$ |
|---|---|---|---|
| Random Forest | MP | 16.5 | 0.874 |
| | MPD | 16.2 | 0.879 |
| | MPDD | 16.4 | 0.876 |
| Extra Trees Regressor | MP | 15.6 | 0.887 |
| | MPD | 14.6 | 0.901 |
| | MPDD | 14.8 | 0.899 |
| Partial Least Squares | MP | 25.1 | 0.71 |
| | MPD | 24.9 | 0.715 |
| | MPDD | 24.8 | 0.715 |
| Lasso Regression | MP | 25.8 | 0.684 |
| | MPD | 22.0 | 0.775 |
| | MPDD | 22.2 | 0.771 |
| Support Vector Regression | MP | 30.0 | 0.589 |
| | MPD | 25.3 | 0.705 |
| | MPDD | 25.2 | 0.707 |
| Neural Network | MP | 16.5 | 0.868 |
| | MPD | 16.2 | 0.879 |
| | MPDD | 16.4 | 0.875 |

**Table S9.** Random-split results (all data): Disintegration Time. Mean Test RMSE and Test $R^2$ over 50 repeated random-split trials are shown for each model and feature set (MP, materials + processes; MPD, MP + scalar descriptors; MPDD, MPD + PSD summary features).

| Model | Feature set | Test RMSE (min) | Test $R^2$ |
|---|---|---|---|
| Random Forest | MP | 7.32 | 0.776 |
| | MPD | 7.57 | 0.760 |
| | MPDD | 7.31 | 0.776 |
| Extra Trees Regressor | MP | 6.66 | 0.809 |
| | MPD | 6.49 | 0.819 |
| | MPDD | 6.80 | 0.801 |
| Partial Least Squares | MP | 9.65 | 0.609 |
| | MPD | 8.70 | 0.684 |
| | MPDD | 8.72 | 0.682 |
| Lasso Regression | MP | 8.97 | 0.662 |
| | MPD | 8.47 | 0.698 |
| | MPDD | 8.39 | 0.704 |
| Support Vector Regression | MP | 12.3 | 0.382 |
| | MPD | 10.0 | 0.589 |
| | MPDD | 10.3 | 0.569 |
| Neural Network | MP | 8.31 | 0.705 |
| | MPD | 7.65 | 0.747 |
| | MPDD | 7.15 | 0.782 |

**Table S10.** Random-split results (all data): Flow function. Mean Test RMSE and Test $R^2$ over 50 repeated random-split trials are shown for each model and feature set (MP, materials + processes; MPD, MP + scalar descriptors; MPDD, MPD + PSD summary features).

| Model | Feature set | Test RMSE (-) | Test $R^2$ |
|---|---|---|---|
| Random Forest | MP | 1.96 | 0.673 |
| | MPD | 1.95 | 0.676 |
| | MPDD | 1.90 | 0.687 |
| Extra Trees Regressor | MP | 1.91 | 0.687 |
| | MPD | 1.86 | 0.700 |
| | MPDD | 1.87 | 0.700 |
| Partial Least Squares | MP | 1.99 | 0.659 |
| | MPD | 1.95 | 0.671 |
| | MPDD | 1.89 | 0.690 |
| Lasso Regression | MP | 2.14 | 0.603 |
| | MPD | 2.22 | 0.569 |
| | MPDD | 2.06 | 0.630 |
| Support Vector Regression | MP | 2.36 | 0.523 |
| | MPD | 2.28 | 0.548 |
| | MPDD | 2.04 | 0.638 |
| Neural Network | MP | 2.48 | 0.399 |
| | MPD | 2.24 | 0.555 |
| | MPDD | 2.18 | 0.585 |

**Table S11.** Random-split results (all data): Cohesion. Mean Test RMSE and Test $R^2$ over 50 repeated random-split trials are shown for each model and feature set (MP, materials + processes; MPD, MP + scalar descriptors; MPDD, MPD + PSD summary features).

| Model | Feature set | Test RMSE (kPa) | Test $R^2$ |
|---|---|---|---|
| Random Forest | MP | 0.117 | 0.722 |
| | MPD | 0.120 | 0.709 |
| | MPDD | 0.116 | 0.724 |
| Extra Trees Regressor | MP | 0.116 | 0.726 |
| | MPD | 0.113 | 0.741 |
| | MPDD | 0.114 | 0.733 |
| Partial Least Squares | MP | 0.122 | 0.690 |
| | MPD | 0.125 | 0.669 |
| | MPDD | 0.122 | 0.687 |
| Lasso Regression | MP | 0.130 | 0.658 |
| | MPD | 0.131 | 0.643 |
| | MPDD | 0.127 | 0.671 |
| Support Vector Regression | MP | 0.131 | 0.650 |
| | MPD | 0.161 | 0.450 |
| | MPDD | 0.137 | 0.607 |
| Neural Network | MP | 0.172 | 0.365 |
| | MPD | 0.183 | 0.318 |
| | MPDD | 0.157 | 0.469 |

**Table S12.** Random-split results (all data): Thickness. Mean Test RMSE and Test $R^2$ over 50 repeated random-split trials are shown for each model and feature set (MP, materials + processes; MPD, MP + scalar descriptors; MPDD, MPD + PSD summary features).

| Model | Feature set | Test RMSE (mm) | Test $R^2$ |
|---|---|---|---|
| Random Forest | MP | 0.0454 | 0.982 |
| | MPD | 0.0443 | 0.984 |
| | MPDD | 0.0489 | 0.980 |
| Extra Trees Regressor | MP | 0.0442 | 0.980 |
| | MPD | 0.0389 | 0.983 |
| | MPDD | 0.0384 | 0.983 |
| Partial Least Squares | MP | 0.115 | 0.892 |
| | MPD | 0.118 | 0.887 |
| | MPDD | 0.113 | 0.895 |
| Lasso Regression | MP | 0.100 | 0.920 |
| | MPD | 0.106 | 0.912 |
| | MPDD | 0.107 | 0.910 |
| Support Vector Regression | MP | 0.0817 | 0.947 |
| | MPD | 0.108 | 0.905 |
| | MPDD | 0.108 | 0.908 |
| Neural Network | MP | 0.385 | −0.299 |
| | MPD | 0.369 | −0.495 |
| | MPDD | 0.394 | −0.500 |

### *S6.2. Time-Split Evaluation Results (All Data)*

This section summarizes the rolling-origin time-split evaluation results aggregated over folds. For each target property, we report Test Spearman's rank correlation, Test RMSE, and Test $R^2$.

**Table S13.** Time-split results (all data): Hardness. Mean Test Spearman's rank correlation, Test RMSE, and Test $R^2$ over 5 rolling-origin folds are shown for each model and feature set (MP, materials + processes; MPD, MP + scalar descriptors; MPDD, MPD + PSD summary features).

| Model | Feature set | Test Spearman | Test RMSE (N) | Test $R^2$ |
|---|---|---|---|---|
| Random Forest | MP | 0.752 | 15.9 | 0.380 |
| | MPD | 0.809 | 14.0 | 0.521 |
| | MPDD | 0.814 | 14.3 | 0.505 |
| Extra Trees Regressor | MP | 0.705 | 16.7 | 0.314 |
| | MPD | 0.801 | 14.7 | 0.471 |
| | MPDD | 0.818 | 14.4 | 0.493 |
| Partial Least Squares | MP | 0.723 | 22.9 | −0.522 |
| | MPD | 0.745 | 21.0 | −0.253 |
| | MPDD | 0.758 | 21.4 | −0.251 |
| Lasso Regression | MP | 0.519 | 26.0 | −1.14 |
| | MPD | 0.387 | 26.7 | −0.864 |
| | MPDD | 0.323 | 24.6 | −0.535 |
| Support Vector Regression | MP | 0.614 | 20.2 | 0.00485 |
| | MPD | 0.755 | 16.3 | 0.285 |
| | MPDD | 0.734 | 18.9 | −0.0185 |

**Table S14.** Time-split results (all data): Disintegration Time. Mean Test Spearman's rank correlation, Test RMSE, and Test $R^2$ over 5 rolling-origin folds are shown for each model and feature set (MP, materials + processes; MPD, MP + scalar descriptors; MPDD, MPD + PSD summary features).

| Model | Feature set | Test Spearman | Test RMSE (min) | Test $R^2$ |
|---|---|---|---|---|
| Random Forest | MP | 0.632 | 9.65 | 0.0476 |
| | MPD | 0.724 | 10.2 | −0.375 |
| | MPDD | 0.656 | 9.66 | −0.0920 |
| Extra Trees Regressor | MP | 0.560 | 9.19 | 0.202 |
| | MPD | 0.61 | 9.04 | 0.191 |
| | MPDD | 0.525 | 9.40 | 0.172 |
| Partial Least Squares | MP | 0.597 | 10.2 | −0.126 |
| | MPD | 0.452 | 10.9 | −0.388 |
| | MPDD | 0.425 | 11.1 | −0.474 |
| Lasso Regression | MP | 0.718 | 8.01 | 0.408 |
| | MPD | 0.564 | 14.8 | −2.34 |
| | MPDD | 0.551 | 14.2 | −2.03 |
| Support Vector Regression | MP | 0.650 | 10.4 | −0.0235 |
| | MPD | 0.441 | 9.91 | −0.00271 |
| | MPDD | 0.0528 | 11.6 | −0.533 |

**Table S15.** Time-split results (all data): Flow function. Mean Test Spearman's rank correlation, Test RMSE, and Test $R^2$ over 5 rolling-origin folds are shown for each model and feature set (MP, materials + processes; MPD, MP + scalar descriptors; MPDD, MPD + PSD summary features).

| Model | Feature set | Test Spearman | Test RMSE (-) | Test $R^2$ |
|---|---|---|---|---|
| Random Forest | MP | 0.518 | 1.99 | 0.281 |
| | MPD | 0.504 | 2.09 | 0.200 |
| | MPDD | 0.526 | 2.10 | 0.178 |
| Extra Trees Regressor | MP | 0.554 | 2.07 | 0.128 |
| | MPD | 0.586 | 2.10 | 0.136 |
| | MPDD | 0.532 | 2.18 | 0.108 |
| Partial Least Squares | MP | 0.546 | 2.44 | −0.0951 |
| | MPD | 0.541 | 2.34 | −0.151 |
| | MPDD | 0.51 | 2.35 | −0.0327 |
| Lasso Regression | MP | 0.531 | 2.49 | −0.206 |
| | MPD | 0.398 | 2.75 | −0.418 |
| | MPDD | 0.510 | 2.26 | 0.0208 |
| Support Vector Regression | MP | 0.574 | 2.22 | −0.00980 |
| | MPD | 0.448 | 2.61 | −0.353 |
| | MPDD | 0.505 | 2.14 | 0.136 |

**Table S16.** Time-split results (all data): Cohesion. Mean Test Spearman's rank correlation, Test RMSE, and Test $R^2$ over 5 rolling-origin folds are shown for each model and feature set (MP, materials + processes; MPD, MP + scalar descriptors; MPDD, MPD + PSD summary features).

| Model | Feature set | Test Spearman | Test RMSE (kPa) | Test $R^2$ |
|---|---|---|---|---|
| Random Forest | MP | 0.600 | 0.135 | 0.660 |
| | MPD | 0.560 | 0.148 | 0.570 |
| | MPDD | 0.597 | 0.143 | 0.594 |
| Extra Trees Regressor | MP | 0.626 | 0.130 | 0.643 |
| | MPD | 0.639 | 0.139 | 0.609 |
| | MPDD | 0.614 | 0.141 | 0.593 |
| Partial Least Squares | MP | 0.669 | 0.142 | 0.565 |
| | MPD | 0.637 | 0.163 | 0.41 |
| | MPDD | 0.637 | 0.161 | 0.448 |
| Lasso Regression | MP | 0.652 | 0.146 | 0.565 |
| | MPD | 0.491 | 0.210 | 0.128 |
| | MPDD | 0.634 | 0.164 | 0.441 |
| Support Vector Regression | MP | 0.669 | 0.156 | 0.472 |
| | MPD | 0.485 | 0.229 | −0.0702 |
| | MPDD | 0.594 | 0.188 | 0.229 |

**Table S17.** Time-split results (all data): Thickness. Mean Test Spearman's rank correlation, Test RMSE, and Test $R^2$ over 5 rolling-origin folds are shown for each model and feature set (MP, materials + processes; MPD, MP + scalar descriptors; MPDD, MPD + PSD summary features).

| Model | Feature set | Test Spearman | Test RMSE (mm) | Test $R^2$ |
|---|---|---|---|---|
| Random Forest | MP | 0.700 | 0.237 | −0.139 |
| | MPD | 0.677 | 0.267 | −0.456 |
| | MPDD | 0.709 | 0.275 | −0.561 |
| Extra Trees Regressor | MP | 0.558 | 0.242 | −0.192 |
| | MPD | 0.530 | 0.303 | −0.792 |
| | MPDD | 0.646 | 0.313 | −0.807 |
| Partial Least Squares | MP | 0.596 | 0.327 | −1.04 |
| | MPD | 0.557 | 0.303 | −0.631 |
| | MPDD | 0.736 | 0.314 | −0.798 |
| Lasso Regression | MP | 0.844 | 0.226 | 0.122 |
| | MPD | 0.577 | 0.319 | −0.897 |
| | MPDD | 0.856 | 0.327 | −1.17 |
| Support Vector Regression | MP | 0.467 | 0.299 | −0.766 |
| | MPD | 0.550 | 0.341 | −0.960 |
| | MPDD | 0.457 | 0.388 | −1.59 |

## *S6.3. Additional Representative Visualizations*

This section provides target-specific visualizations that support the results summarized in the main text.

### S6.3.1. Representative Parity Plots for the Best Models

The following parity plots show one representative random split for the best model (algorithm + feature set) for each target variable. Both Train+Dev and Test predictions are shown in each plot.

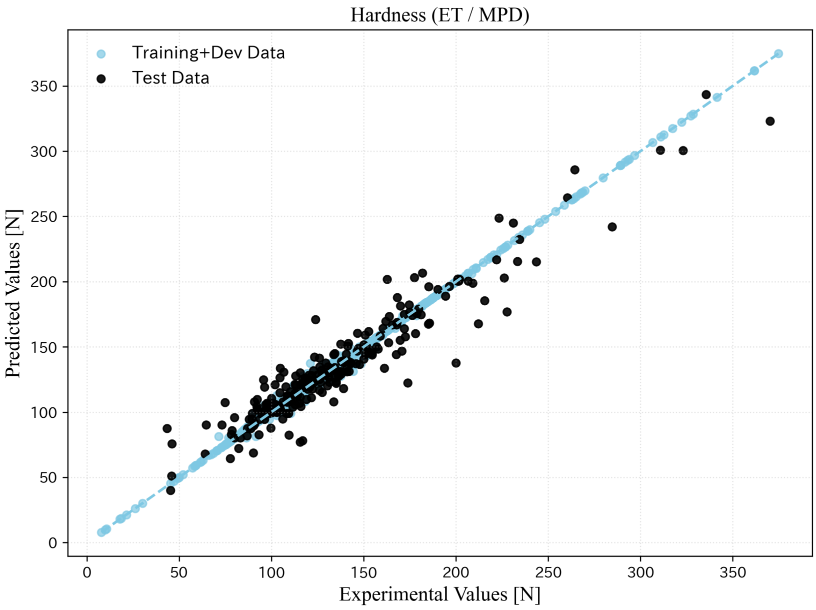


**Figure S1.** Representative parity plot for the best model on the test dataset (Hardness, ET, MPD). Points show Train+Dev and Test predictions for one representative random split.

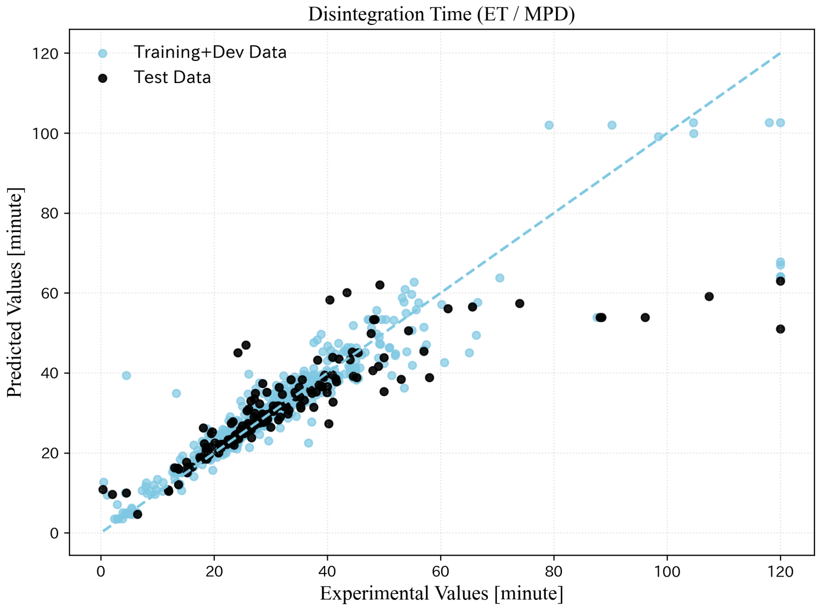


**Figure S2.** Representative parity plot for the best model on the test dataset (Disintegration Time, ET, MPD). Points show Train+Dev and Test predictions for one representative random split.

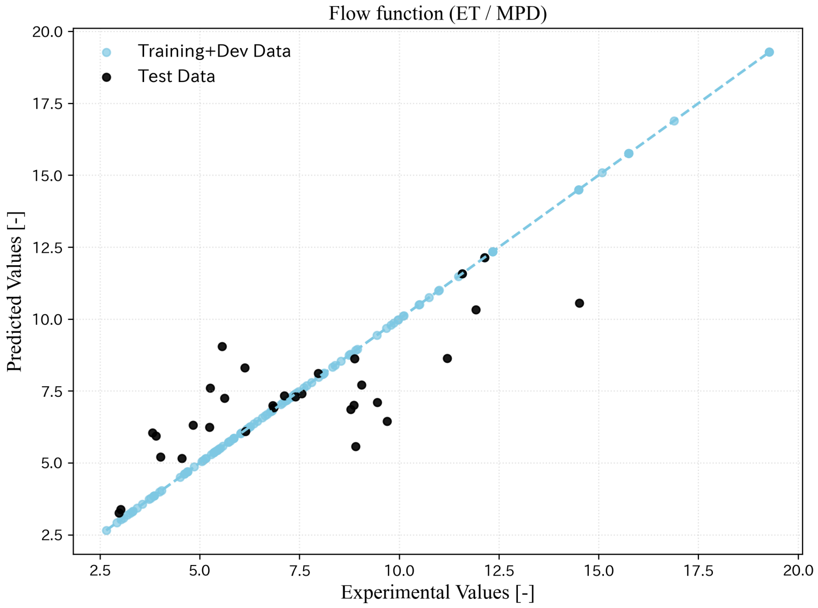


**Figure S3.** Representative parity plot for the best model on the test dataset (Flow function, ET, MPD). Points show Train+Dev and Test predictions for one representative random split.

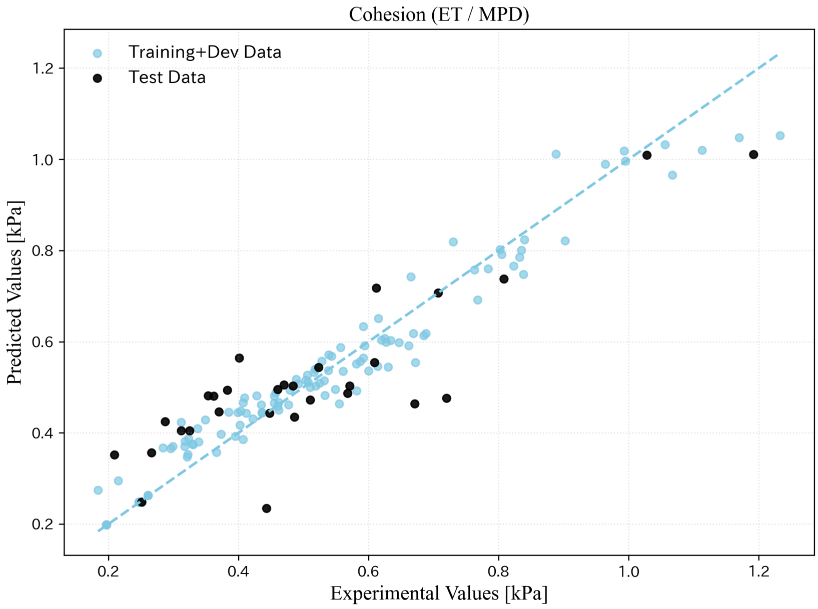


**Figure S4.** Representative parity plot for the best model on the test dataset (Cohesion, ET, MPD). Points show Train+Dev and Test predictions for one representative random Split.

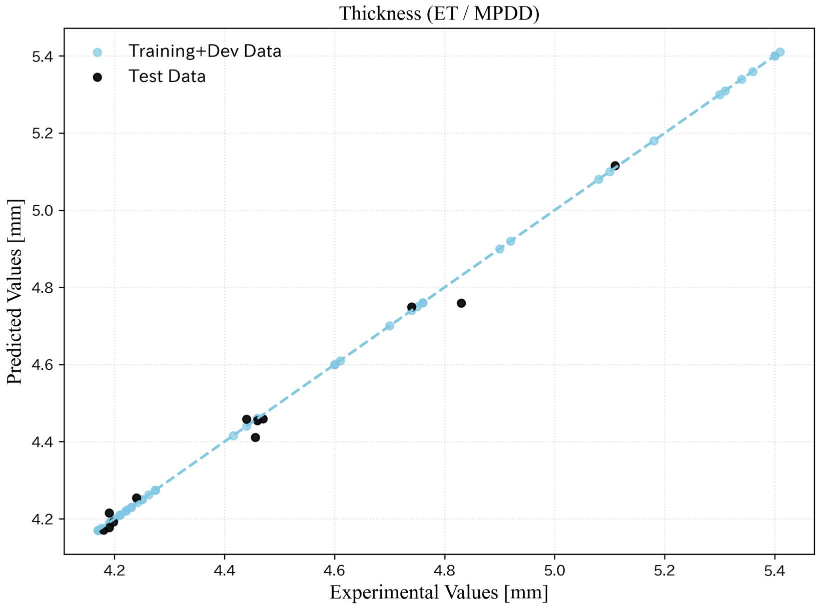


**Figure S5.** Representative parity plot for the best model on the test dataset (Thickness, ET, MPDD). Points show Train+Dev and Test predictions for one representative random Split.

### S6.3.2. Additional SHAP Importance Plots

Representative SHAP importance plots for Flow function, Cohesion, and Thickness are provided here as supplementary visualizations. The main text retains the corresponding SHAP plots for Hardness and Disintegration Time.

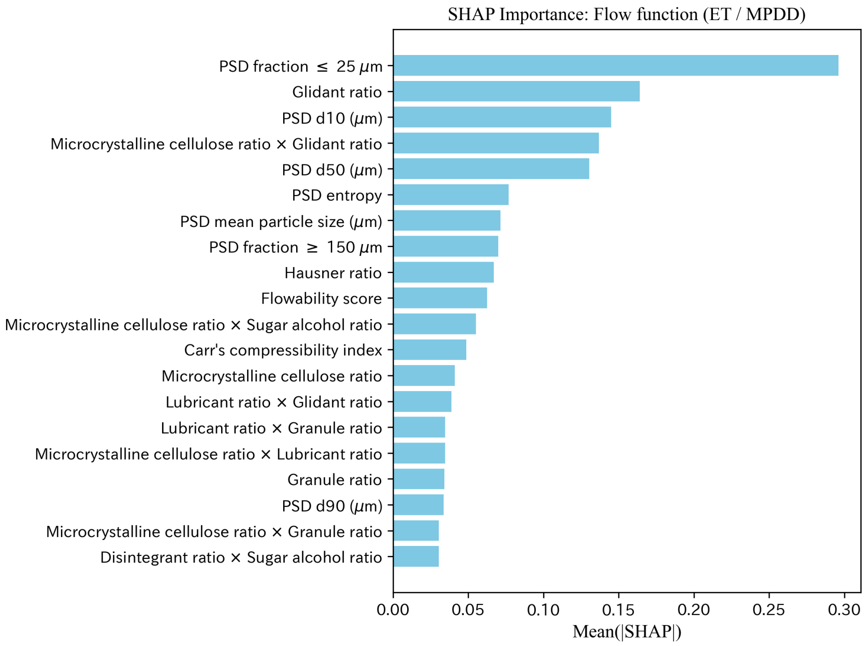


**Figure S6.** SHAP feature importance (Mean(|SHAP|)): Flow function (ET/MPDD). Bars show mean absolute SHAP values aggregated over 50 repeated random splits.

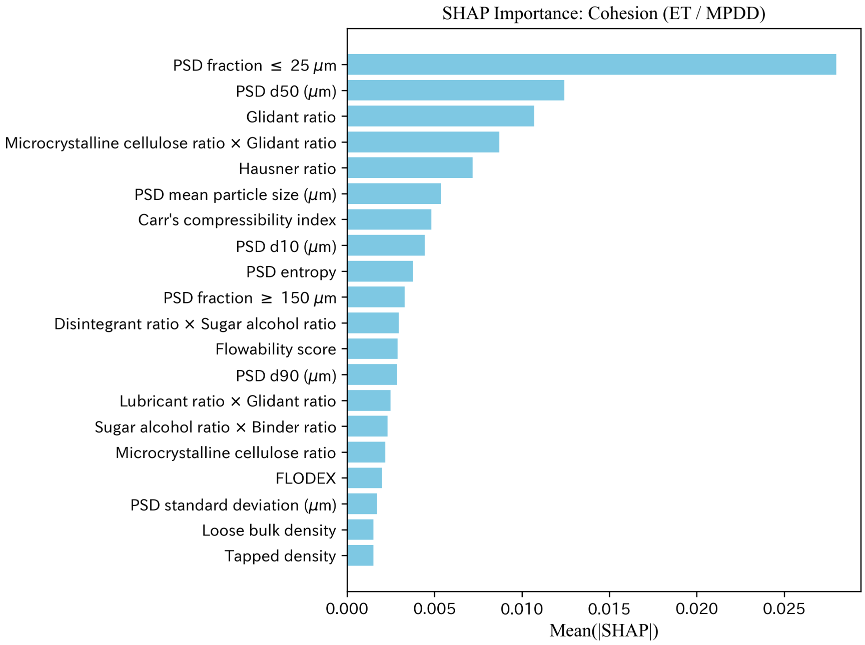


**Figure S7.** SHAP feature importance (Mean(|SHAP|)): Cohesion (ET/MPDD). Bars show mean absolute SHAP values aggregated over 50 repeated random splits.

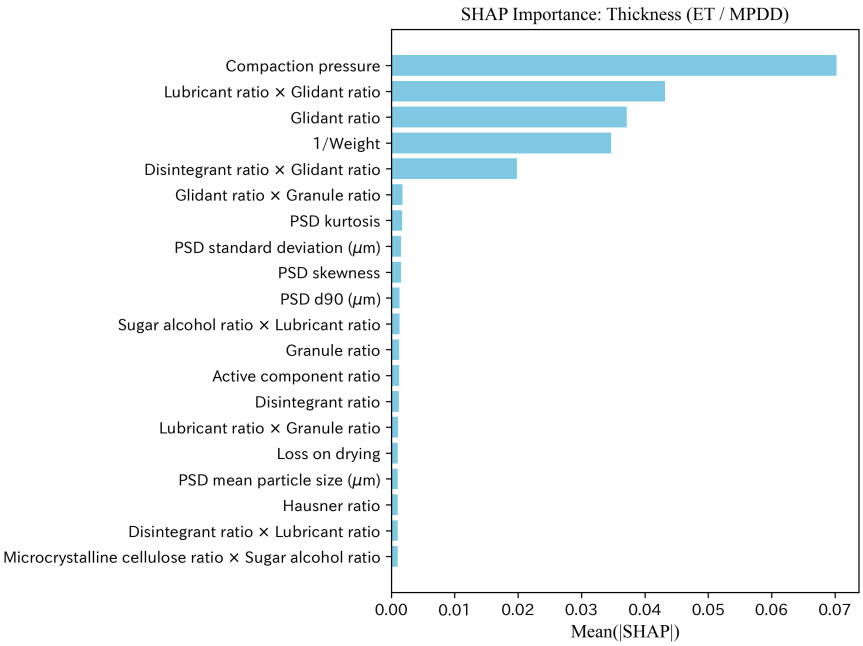


**Figure S8.** SHAP feature importance (Mean(|SHAP|)): Thickness (ET/MPDD). Bars show mean absolute SHAP values aggregated over 50 repeated random splits.

*S6.4. Covariate-Shift Analysis Details (JSD as Primary; PSI as Supplementary)*

In the main manuscript, covariate shift is characterized using JSD on non-missing feature distributions. As a supplementary reference, we also report Population Stability Index (PSI) summaries and representative PSI-based top-20 feature plots here.

JSD specification (main manuscript).

For each feature, we computed Jensen–Shannon divergence (JSD) between the Train+Dev distribution and the Test distribution using non-missing values only. Under rolling-origin splitting, JSD was computed separately for each fold (Train+Dev vs Test within that fold) and then aggregated by averaging the per-fold JSD values for each feature. Features were ranked by this fold-averaged JSD, and the reported mean JSD (Top 10) corresponds to the mean of the top-10 fold-averaged JSD values. In this study, the later-period formulation groups also included conditions with higher granule usage ratios and different proportions of some mineral-based raw materials; these formulation changes are considered plausible contributors to the observed temporal covariate shift, especially for Disintegration Time. Given discrete probability vectors $p$ and $q$ over bins, we use the standard definition

$$\mathrm{JSD}(p,q) = \tfrac{1}{2}\mathrm{KL}(p\|m) + \tfrac{1}{2}\mathrm{KL}(q\|m), \quad m = (p+q)/2,$$

with base-2 logarithms ($\log_2$), so that $\mathrm{JSD} \in [0,1]$. Continuous variables were discretized into 10 bins using quantile-based bin edges fitted on Train+Dev (equal-frequency on Train+Dev); when Train+Dev was (near-)constant, we fell back to equal-width bins using the combined min/max of Train+Dev and Test. To avoid numerical issues with empty bins, we applied additive smoothing to the binned counts using $\epsilon = 10^{-12}$ (added to each bin before normalization). Missing values (NaN/inf) were excluded from the non-missing JSD computation, and missing-rate shift was summarized separately by the absolute difference in missing rate and by a two-point JSD over $\{\text{missing}, \text{non-missing}\}$. To address the project-specific convention that some PSD-derived features may contain zero-coded placeholders,

we treated 0 as missing only for PSD summary features; for other features, 0 was treated as a valid numeric value.

Additional JSD top-20 plots for MP and MPDD (supplementary).

Because the main manuscript shows representative top-20 JSD bar plots for MPD, we additionally provide MP and MPDD versions here for completeness. These supplementary plots allow direct confirmation of how top-shifted features change when moving from MP to MPDD, including cases where PSD-derived summary features become dominant in MPDD.

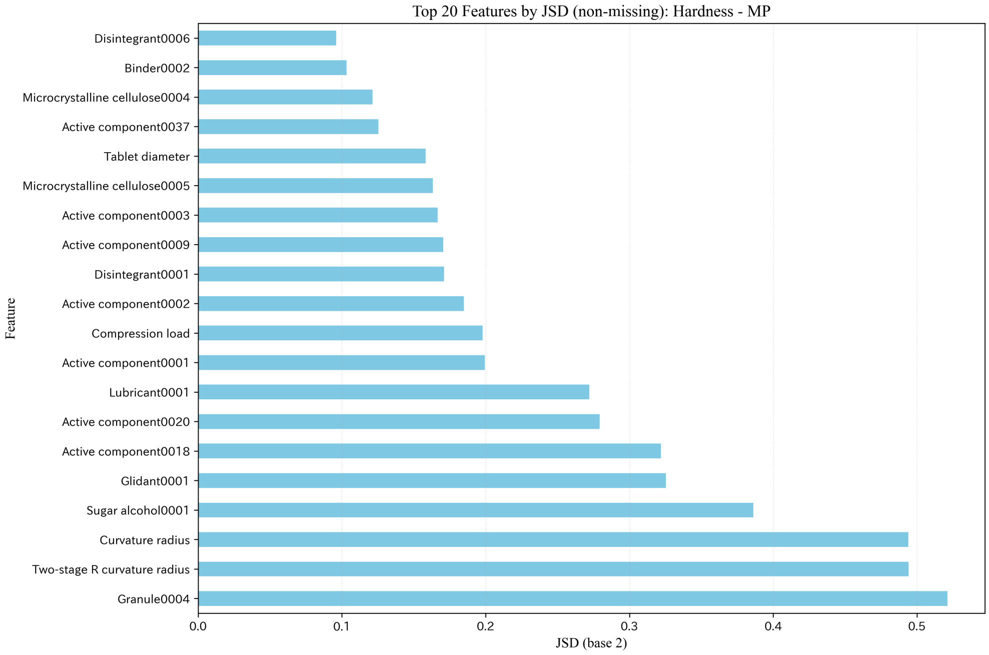


**Figure S9.** Top 20 features by JSD under the rolling-origin split (Hardness, MP). Bars show fold-averaged Jensen–Shannon divergence between Train+Dev and Test distributions computed on non-missing values only; larger values indicate stronger covariate shift.

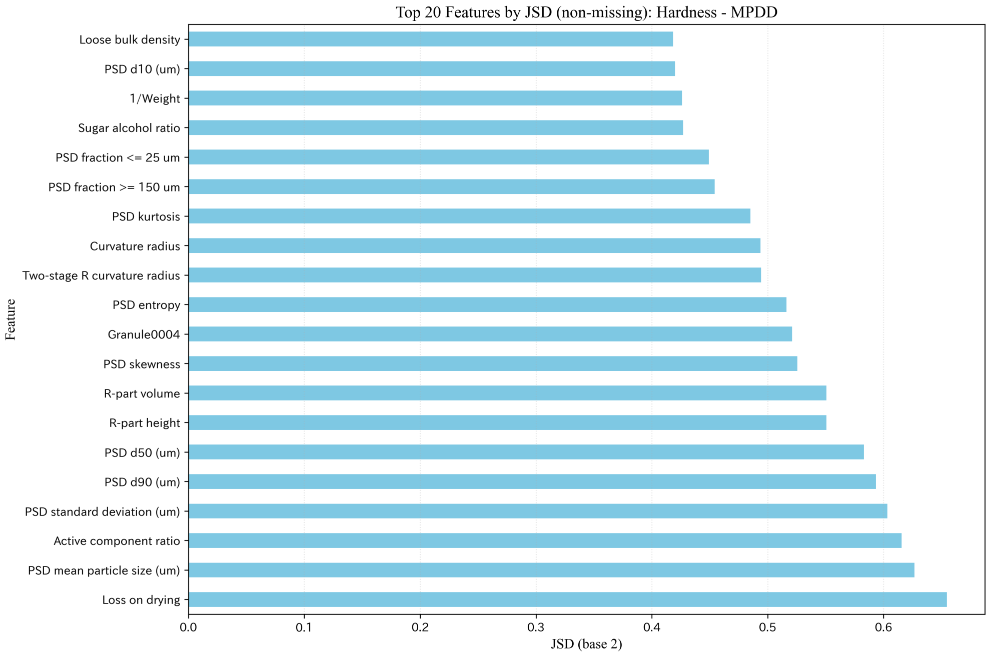


**Figure S10.** Top 20 features by JSD under the rolling-origin split (Hardness, MPDD). Bars show fold-averaged Jensen–Shannon divergence between Train+Dev and Test distributions computed on non-missing values only; larger values indicate stronger covariate shift.

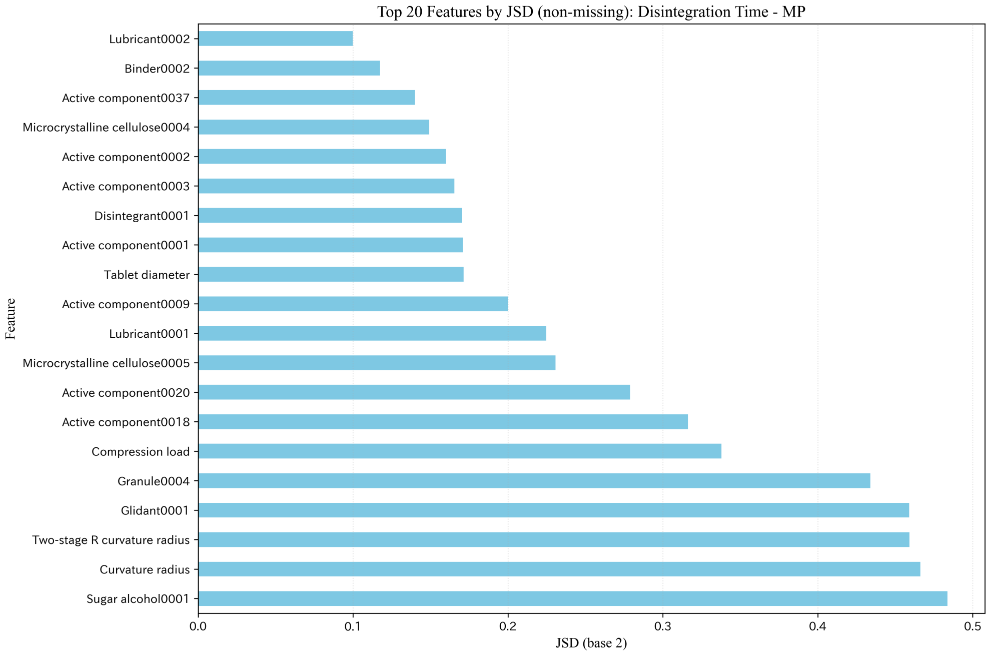


**Figure S11.** Top 20 features by JSD under the rolling-origin split (Disintegration Time, MP). Bars show fold-averaged Jensen–Shannon divergence between Train+Dev and Test distributions computed on non-missing values only; larger values indicate stronger covariate shift.

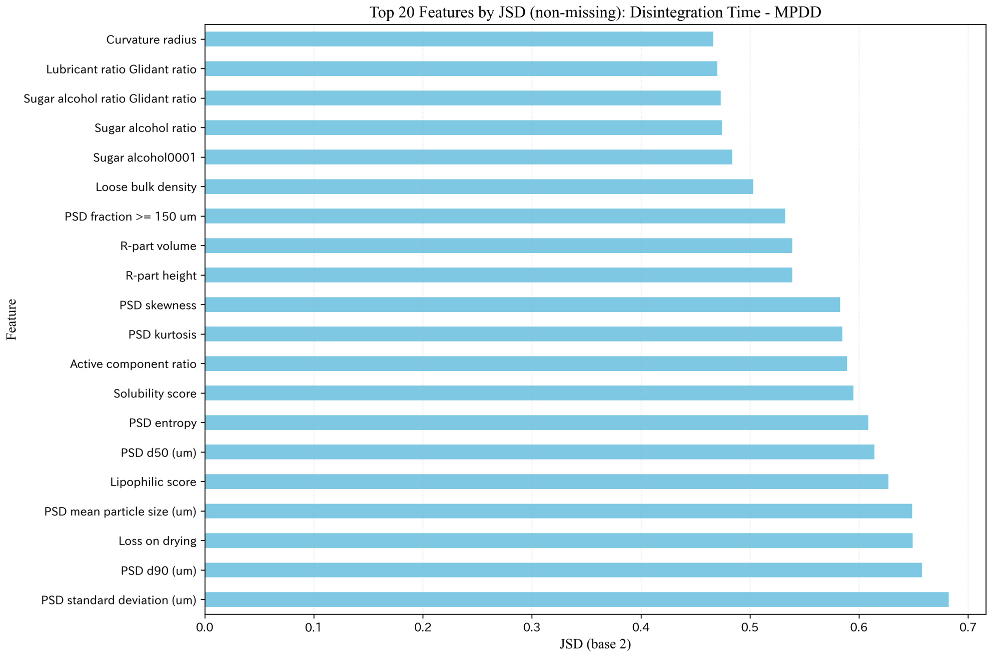


**Figure S12.** Top 20 features by JSD under the rolling-origin split (Disintegration Time, MPDD). Bars show fold-averaged Jensen–Shannon divergence between Train+Dev and Test distributions computed on non-missing values only; larger values indicate stronger covariate shift.

PSI specification (supplementary reference).

PSI was computed with 10 equal-width bins defined on the combined min/max range of Train+Dev and Test for each feature. Let $p_b$ and $q_b$ denote the smoothed bin probabilities of Train+Dev and Test, respectively; we used

$$\mathrm{PSI} = \sum_b (q_b - p_b) \ln\left(\frac{q_b}{p_b}\right),$$

with natural logarithms. To handle bins with zero frequency, we added $\epsilon = 10^{-10}$ to each bin count before normalization to avoid zero probabilities.

**Table S18.** PSI summary of top-10 shifted features under rolling-origin split. Mean PSI (Top 10) and Max PSI (Top 10) are calculated from the top-10 fold-averaged PSI features within each target and feature set; larger values indicate stronger covariate shift.

| Target | Feature set | Mean PSI (Top 10) | Max PSI (Top 10) |
|---|---|---|---|
| Hardness | MP | 15.41 | 29.28 |
| | MPD | 22.94 | 33.71 |
| | MPDD | 24.03 | 33.71 |
| Disintegration Time | MP | 15.63 | 22.70 |
| | MPD | 22.93 | 29.32 |
| | MPDD | 24.45 | 29.32 |

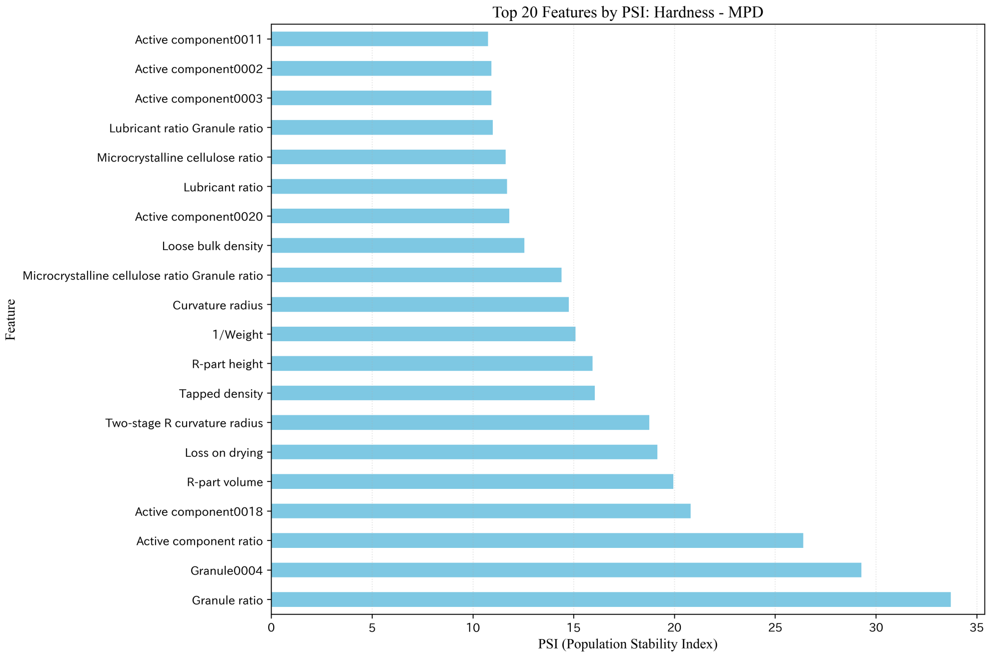


**Figure S13.** Top 20 features by PSI under the rolling-origin split (Hardness, MPD). Bars show fold-averaged Population Stability Index between Train+Dev and Test distributions; larger values indicate stronger covariate shift.

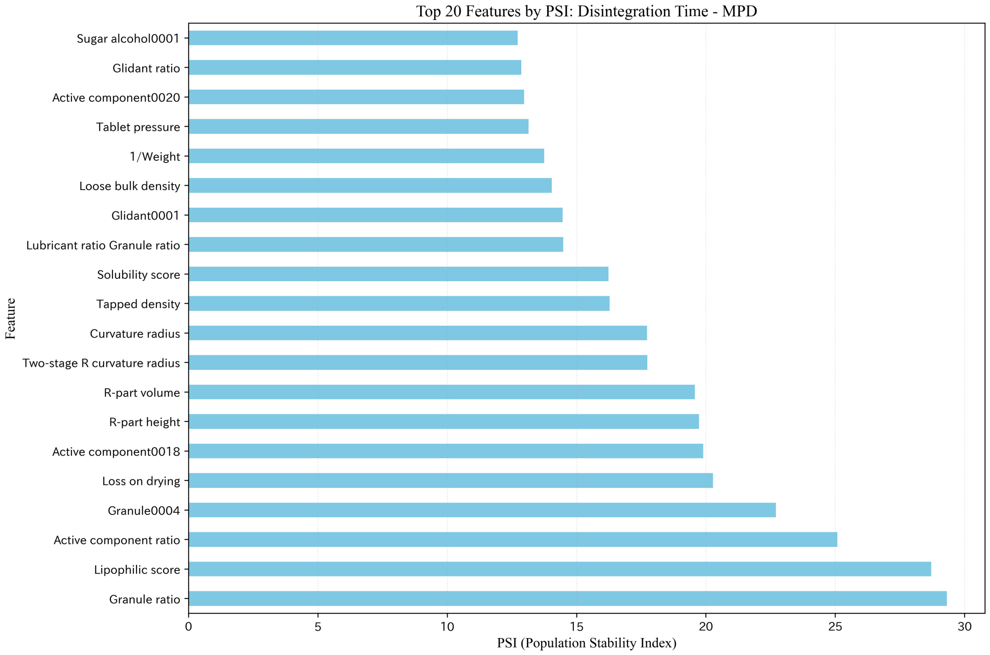


**Figure S14.** Top 20 features by PSI under the rolling-origin split (Disintegration Time, MPD). Bars show fold-averaged Population Stability Index between Train+Dev and Test distributions; larger values indicate stronger covariate shift.

## S7. Request Procedure for Materials/Code and Restricted Data

Full formulation records and the full source code are not publicly disclosed in this Supporting Information due to confidentiality constraints in proprietary product development. They are available from the corresponding author upon reasonable request, subject to confidentiality considerations (e.g., a non-disclosure agreement, as applicable).
